# Scheduling to Minimize Age of Incorrect Information with Imperfect Channel State Information

**DOI:** 10.3390/e23121572

**Published:** 2021-11-25

**Authors:** Yutao Chen, Anthony Ephremides

**Affiliations:** Department of Electrical and Computer Engineering, University of Maryland, College Park, MD 20742, USA; cheny@umd.edu

**Keywords:** age of incorrect information, multi-user system, scheduling policy

## Abstract

In this paper, we study a slotted-time system where a base station needs to update multiple users at the same time. Due to the limited resources, only part of the users can be updated in each time slot. We consider the problem of minimizing the Age of Incorrect Information (AoII) when imperfect Channel State Information (CSI) is available. Leveraging the notion of the Markov Decision Process (MDP), we obtain the structural properties of the optimal policy. By introducing a relaxed version of the original problem, we develop the Whittle’s index policy under a simple condition. However, indexability is required to ensure the existence of Whittle’s index. To avoid indexability, we develop Indexed priority policy based on the optimal policy for the relaxed problem. Finally, numerical results are laid out to showcase the application of the derived structural properties and highlight the performance of the developed scheduling policies.

## 1. Introduction

The Age of Incorrect Information (AoII) is introduced in [1] as a combination of age-based metrics (e.g., Age of Information (AoI)) and error-based metrics (e.g., Minimum Mean Square Error). In communication systems, AoII captures not only the information mismatch between the source and the destination but also the aging process of inconsistent information. Hence, two functions dominate AoII. The first is the time penalty function, which reflects how the inconsistency of information affects the system over time. In real-life applications, inconsistent information will affect different communication systems in different ways. For example, machine temperature monitoring is time-sensitive because the damage caused by overheating will accumulate quickly. However, reservoir water level monitoring is less sensitive to time. Therefore, by adopting different time penalty functions, AoII can capture different aging processes of the mismatch in different systems. The second is the information penalty function, which captures the information mismatch between the source and the destination. It allows us to measure mismatches in different ways, depending on how sensitive different systems are to information inconsistencies. For example, the navigation system requires precise information to give correct instructions, but the real-time delivery tracking system does not need very accurate location information. Since we can choose different penalty functions for different systems, AoII is adaptable to various communication goals, which is why it is regarded as a semantic metric [2].

Since the introduction of AoII, several studies have been performed to reveal its fundamental nature. The authors of [3] consider a system with random packet delivery times and compare AoII with AoI and real-time error via extensive numerical results. The authors of [4] study the problem of minimizing the AoII that takes the general time penalty function. Three real-life applications are considered to showcase the performance advantages of AoII over AoI and real-time error. In [5], the authors investigate the AoII that considers the quantified mismatch between the source and the destination. The optimization problem is studied when the system is resource-constrained. The authors of [6] studied the AoII minimization problem in the context of scheduling. It considers a system where the central scheduler needs to update multiple users at the same time. However, the central scheduler cannot know the states of the sources before receiving the updates. By introducing the belief value, Whittle’s index policy is developed and evaluated. In this paper, we also consider the problem of minimizing AoII in scheduling. Different from [6], we consider the generic time penalty function and study the minimization problem in the presence of imperfect Channel State Information (CSI). Due to the existence of CSI, Whittle’s index policy becomes infeasible in general. Hence, we introduce another scheduling policy that is more versatile and has comparable performance to Whittle’s index policy.

The problem of scheduling to minimize AoI is studied under various system settings in [7,8,9,10,11]. The problem studied in this paper is different and more complicated because AoII considers the aging process of inconsistent information rather than the aging process of updates. Meanwhile, none of them consider the case where CSI is available. The problem of optimizing information freshness in the presence of CSI is studied in [12,13]. However, they focus on the system with a single user and mainly discuss the case where CSI is perfect. The scheduling problems with the goal of minimizing an error-based performance measure are considered in [14,15,16]. Our problem is fundamentally different because AoII also considers the time effect. Moreover, we consider the system where a base station observes multiple sources simultaneously and needs to send updates to multiple destinations.

The main contributions of this work can be summarized as follows. (1) We study the problem of minimizing AoII in a multi-user system where imperfect CSI is available. Meanwhile, the time penalty function is generic. (2) We derive the structural properties of the optimal policy for the considered problem. (3) We establish the indexability of the considered problem under a simple condition and develop Whittle’s index policy. (4) We obtain the optimal policy for a relaxed version of the original problem. By exploring the characteristics of the relaxed problem, we provide an efficient algorithm to obtain the optimal policy. (5) Based on the optimal policy for the relaxed problem, we develop the Indexed priority policy that is free from indexability and has comparable performance to Whittle’s index policy.

The remainder of this paper is organized in the following way. In Section 2, we introduce the system model and formulate the primal problem. Section 3 explores the structural properties of the optimal policy for the primal problem. Under a simple condition, we develop Whittle’s index policy in Section 4. Section 5 presents the optimal policy for a relaxed version of the primal problem. On this basis, we develop the Indexed priority policy in Section 6. Finally, in Section 7, the numerical results are laid out.

## 2. System Overview

### 2.1. Communication Model

**Figure 1:**
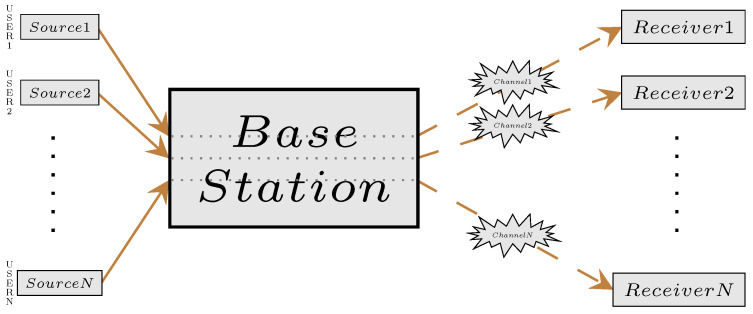
The structure of the communication model.

We consider a slotted-time system with *N* users and one base station. Each user is composed of a source process, a channel, and a receiver. We assume all the users share the same structure, but the parameters are different. The structure of the communication model is provided in Figure 1.

For user *i*, the source process is modeled by a two-state Markov chain where transitions happen between the two states with probability $p_{i}>0$ and self-transitions happen with probability $1-p_{i}$. At any time slot *t*, the state of the source process $X_{i,t}\in \left\{0,1\right\}$ will be reported to the base station as an update, and the base station will decide whether to transmit this update through the corresponding channel. The channel is unreliable, but the estimate of the Channel State Information (CSI) is available at the beginning of each time slot. Let $r_{i,t}\in \left\{0,1\right\}$ be the CSI at time *t*. We assume that $r_{i,t}$ is independent across time and user indices. $r_{i,t}=1$ if and only if the transmission attempt at time *t* will succeed and $r_{i,t}=0$ otherwise. Then, we denote by ${\hat{r}}_{i,t}\in \left\{0,1\right\}$ the estimate of $r_{i,t}$. We assume that ${\hat{r}}_{i,t}$ is an independent Bernoulli random variable with parameter $\gamma_{i}$, i.e., ${\hat{r}}_{i,t}=1$ with probability $\gamma_{i}\in \left[0,1\right]$ and ${\hat{r}}_{i,t}=0$ with probability $1-\gamma_{i}$. However, the estimate is imperfect. We assume that the error depends only on the user and its estimate. More precisely, we define the probability of error as $p_{e,i}^{{\hat{r}}_{i}}≜Pr\left[r_{i}\neq {\hat{r}}_{i}|{\hat{r}}_{i}\right]$. We assume $p_{e,i}^{{\hat{r}}_{i}}<0.5$ because we can flip the estimate if $p_{e,i}^{{\hat{r}}_{i}}>0.5$. We are not interested in the case of $p_{e,i}^{{\hat{r}}_{i}}=0.5$ since ${\hat{r}}_{i,t}$ is useless in this case. Although the channel is unreliable, each transmission attempt takes exactly one time slot regardless of the result, and the successfully transmitted update will not be corrupted. Every time an update is received, the receiver will use it as the new estimate ${\hat{X}}_{i,t}$. The receiver will send an ACK/NACK packet to inform the base station of its reception of the new update. Since an ACK/NACK packet is generally very small and simple, we assume that it is transmitted reliably and received instantaneously. Then, if ACK is received, the base station knows that the receiver’s estimate changed to the transmitted update. If NACK is received, the base station knows that the receiver’s estimate did not change. Therefore, the base station always knows the estimate at the receiver side.

At the beginning of each time slot, the base station receives updates from each source and the estimates of CSI from each channel. The old updates and estimates are discarded upon the arrival of new ones. Then, the base station decides which updates to transmit, and the decision is independent of the transmission history. Due to the limited resources, at most M<N updates are allowed per transmission attempt. We consider a base station that always transmits *M* updates.

### 2.2. Age of Incorrect Information

All the users adopt AoII as a performance metric, but the choices of penalty functions vary. Let $X_{t}$ and ${\hat{X}}_{t}$ be the true state and the estimate of the source process, respectively. Then, in a slotted-time system, AoII can be expressed as follows

(1) $$\Delta_{AoII}\left(X_{t},{\hat{X}}_{t},t\right)=\sum_{k=U_{t}+1}^{t}\left(g\left(X_{k},{\hat{X}}_{k}\right)\times F\left(k-U_{t}\right)\right),$$

where $U_{t}$ is the last time instance before time *t* (including *t*) that the receiver’s estimate is correct. $g(X_{t},{\hat{X}}_{t})$ can be any information penalty function that captures the difference between $X_{t}$ and ${\hat{X}}_{t}$. F(t)≜f(t)−f(t−1) where f(t) can be any time penalty function that is non-decreasing in *t*. We consider the case where the users adopt the same information penalty function $g\left(X_{t},{\hat{X}}_{t}\right)=\left|X_{t}-{\hat{X}}_{t}\right|$ but possibly different time penalty functions. To ease the analysis, we require f(t) to be unbounded. Combined together, we require $f\left(t_{1}\right)\leq f\left(t_{2}\right)$ if $t_{1}<t_{2}$ and $\lim_{t\to +\infty }f\left(t\right)=+\infty$. Without a loss of generality, we assume f(0)=0, as the source is modeled by a two-state Markov chain, $g\left(X_{t},{\hat{X}}_{t}\right)\in \left\{0,1\right\}$. Hence, Equation (1) can be simplified to

$$\Delta_{AoII}\left(X_{t},{\hat{X}}_{t},t\right)=\sum_{k=U_{t}+1}^{t}F\left(k-U_{t}\right)=f\left(s_{t}\right),$$

where $s_{t}≜t-U_{t}$. Therefore, the evolution of $s_{t}$ is sufficient to characterize the evolution of AoII. To this end, we distinguish between the following cases.

- When the receiver’s estimate is correct at time t+1, we have $U_{t+1}=t+1$. Then, by definition, $s_{t+1}=0$.
- When the receiver’s estimate is incorrect at time t+1, we have $U_{t+1}=U_{t}$. Then, by definition, $s_{t+1}=t+1-U_{t}=s_{t}+1$.

To sum up, we get

(2) $$s_{t+1}={𝟙}_{\left\{U_{t+1}\neq t+1\right\}}\times \left(s_{t}+1\right).$$

**Figure 2:**
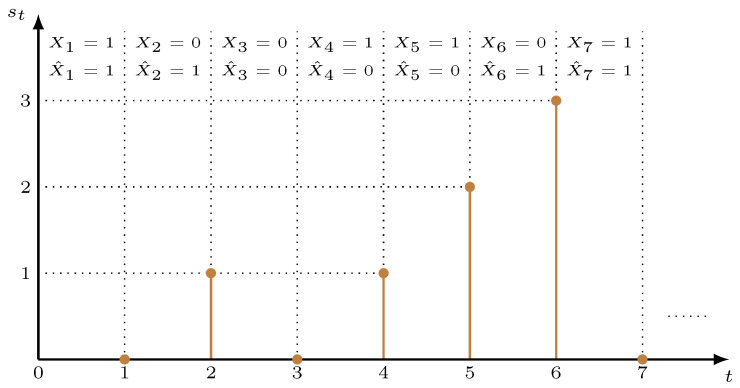
A sample path of $s_{t}$.

A sample path of $s_{t}$ is shown in Figure 2. In the remainder of this paper, we use $f_{i}\left(\cdot \right)$ to denote the time penalty function user *i* adopts.

**Remark** **1.** *Under this particular choice of the penalty function, $s_{t}$ can be interpreted as the time elapsed since the last time the receiver’s estimate is correct. Please note that $s_{t}$ is different from the Age of Information (AoI) [17], which is defined as the time elapsed since the generation time of the last received update. We can see that AoI considers the aging process of the update, while AoII considers the aging process of the estimation error. At the same time, $s_{t}$ is also fundamentally different from the holding time, which, according to [18,19], is defined as the time elapsed since the last successful transmission. We notice that the receiver’s estimate can become correct even when no new update is successfully transmitted. Moreover, the information carried by the update may have become incorrect by the time it is received. We also notice that [18,19] consider the problem of minimizing the estimation error. However, by adopting AoII as the performance metric, we study the impact of estimation error on the system.*

### 2.3. System Dynamic

In this section, we tackle the system dynamic. We notice that the status of user *i* can be captured by the pair $x_{i,t}≜\left(s_{i,t},{\hat{r}}_{i,t}\right)$. In the following, we will use $x_{i,t}$ and $\left(s_{i,t},{\hat{r}}_{i,t}\right)$ interchangeably. Then, the system dynamic can be fully characterized by the dynamic of $x_{t}≜\left(x_{1,t},\ldots ,x_{N,t}\right)$. Hence, it suffices to characterize the value of $x_{t+1}$ given $x_{t}$ and the base station’s action. To this end, we denote, by $a_{t}=\left(a_{1,t},\ldots ,a_{N,t}\right)$, the base station’s action at time *t*. $a_{i,t}=1$ if the base station transmits the update from user *i* at time *t* and $a_{i,t}=0$ otherwise. We notice that given action $a_{t}$, users are independent and the action taken on user *i* will only affect itself. Consequently

$$Pr\left(x_{t+1}|x_{t},a_{t}\right)=\prod_{i=1}^{N}Pr\left(x_{i,t+1}|x_{i,t},a_{t}\right)=\prod_{i=1}^{N}Pr\left(x_{i,t+1}|x_{i,t},a_{i,t}\right).$$

Combined with the fact that all the users share the same structure, it is sufficient to study the dynamic of a single user. In the following discussions, we drop the user-dependent subscript *i*. We recall that ${\hat{r}}_{t+1}$ is an independent Bernoulli random variable. Then, we have

(3) $$Pr\left(x_{t+1}|x_{t},a_{t}\right)=P\left({\hat{r}}_{t+1}\right)\times Pr\left(s_{t+1}|x_{t},a_{t}\right).$$

By definition, $P({\hat{r}}_{t+1}=1)=\gamma$ and $P({\hat{r}}_{t+1}=0)=1-\gamma$. Then, we only need to tackle the value of $Pr(s_{t+1}|x_{t},a_{t})$. To this end, we distinguish between the following cases

- When $x_{t}=\left(0,{\hat{r}}_{t}\right)$, the estimate at time *t* is correct (i.e., ${\hat{X}}_{t}=X_{t}$). Hence, for the receiver, $X_{t}$ carries no new information about the source process. In other words, ${\hat{X}}_{t+1}={\hat{X}}_{t}$ regardless of whether an update is transmitted at time *t*. We recall that $U_{t+1}=U_{t}$ if ${\hat{X}}_{t+1}\neq X_{t+1}$ and $U_{t+1}=t+1$ otherwise. Since the source is binary, we obtain $U_{t+1}=U_{t}$ if $X_{t+1}\neq X_{t}$, which happens with probability *p* and $U_{t+1}=t+1$ otherwise. According to (2), we obtain
  $$Pr(1|\left(0,{\hat{r}}_{t}\right),a_{t})=p,$$
  $$Pr(0|\left(0,{\hat{r}}_{t}\right),a_{t})=1-p.$$
- When $a_{t}=0$ and $x_{t}=\left(s_{t},{\hat{r}}_{t}\right)$, where $s_{t}>0$, the channel will not be used and no new update will be received by the receiver, and so, ${\hat{X}}_{t+1}={\hat{X}}_{t}$. We recall that $U_{t+1}=U_{t}$ if ${\hat{X}}_{t+1}\neq X_{t+1}$ and $U_{t+1}=t+1$ otherwise. Since $X_{t}\neq {\hat{X}}_{t}$ and the source is binary, we have $U_{t+1}=U_{t}$ if $X_{t+1}=X_{t}$, which happens with probability 1−p and $U_{t+1}=t+1$ otherwise. According to (2), we obtain
  $$Pr(s_{t}+1|\left(s_{t},{\hat{r}}_{t}\right),a_{t}=0)=1-p,$$
  $$Pr(0|\left(s_{t},{\hat{r}}_{t}\right),a_{t}=0)=p.$$
- When $a_{t}=1$ and $x_{t}=\left(s_{t},1\right)$ where $s_{t}>0$, the transmission attempt will succeed with probability $1-p_{e}^{1}$ and fail with probability $p_{e}^{1}$. We recall that $U_{t+1}=U_{t}$ if ${\hat{X}}_{t+1}\neq X_{t+1}$ and $U_{t+1}=t+1$ otherwise. Then, when the transmission attempt succeeds (i.e., ${\hat{X}}_{t+1}=X_{t}$), $U_{t+1}=U_{t}$ if $X_{t+1}\neq X_{t}$ and $U_{t+1}=t+1$ otherwise. When the transmission attempt fails (i.e., ${\hat{X}}_{t+1}={\hat{X}}_{t}\neq X_{t}$), we have $U_{t+1}=U_{t}$ if $X_{t+1}=X_{t}$ and $U_{t+1}=t+1$ otherwise. Combining (2) with the dynamic of the source process we obtain
  $$Pr\left(s_{t}+1|\left(s_{t},1\right),a_{t}=1\right)=p_{e}^{1}\left(1-p\right)+\left(1-p_{e}^{1}\right)p≜\alpha ,$$
  $$Pr\left(0|\left(s_{t},1\right),a_{t}=1\right)=p_{e}^{1}p+\left(1-p_{e}^{1}\right)\left(1-p\right)=1-\alpha .$$
- When $a_{t}=1$ and $x_{t}=\left(s_{t},0\right)$, where $s_{t}>0$, following the same line, we obtain
  $$Pr\left(s_{t}+1|\left(s_{t},0\right),a_{t}=1\right)=p_{e}^{0}p+\left(1-p_{e}^{0}\right)\left(1-p\right)≜\beta ,$$
  $$Pr\left(0|\left(s_{t},0\right),a_{t}=1\right)=p_{e}^{0}\left(1-p\right)+\left(1-p_{e}^{0}\right)p=1-\beta .$$

Combines together, we obtain the value of $Pr(s_{t+1}|x_{t},a_{t})$ in all cases. As only *M* out of *N* updates are allowed per transmission attempt, we realize a necessity to require transmission attempts always help minimize AoII. It is equivalent to impose $Pr\left(s_{t+1}>s_{t}|\left(s_{t},{\hat{r}}_{t}\right),a_{t}=0\right)>Pr\left(s_{t+1}>s_{t}|\left(s_{t},{\hat{r}}_{t}\right),a_{t}=1\right)$ for any $\left(s_{t},{\hat{r}}_{t}\right)$. Leveraging the results above, it is sufficient to require p<0.5. As all the users share the same structure, we assume, for the rest of this paper, that $0<p_{i}<0.5$ for 1≤i≤N.

### 2.4. Problem Formulation

The communication goal is to minimize the expected AoII. Therefore, the problem can be formulated as the following

(4a)arg minϕ ∈ ΦlimT→∞1TEϕ(∑t=0T−1∑i=1Nfi(si,t))(4b)subject to∑i=1Nai,t = M ∀t,

where Φ is the set of all causal policies. We refer to the constrained minimization problem reported in problem (4) as the Primal Problem (PP). We notice that the PP is a Restless Multi-Armed Bandit (RMAB) Problem. The optimal policy for this type of problem is far from reachable since it is PSPACE-hard in general [20]. However, we can still derive the structural properties of the optimal policy. These structural properties can be used as a guide for the development of scheduling policies and can indicate the good performance of the developed scheduling policies.

## 3. Structural Properties of the Optimal Policy

In this section, we investigate the structural properties of the optimal policy for PP. We first define an infinite horizon with an average cost Markov Decision Process (MDP) $M_{N}\left(w,M\right)=\left(X_{N},A_{N}\left(M\right),P_{N},C_{N}\left(w\right)\right)$, where

- $X_{N}$ denotes the state space. The state is $x=(x_{1},\ldots ,x_{N})$ where $x_{i}=\left(s_{i},{\hat{r}}_{i}\right)$.
- $A_{N}\left(M\right)$ denotes the action space. The feasible action is $a=(a_{1},\ldots ,a_{N})$ where $a_{i}\in \left\{0,1\right\}$ and $\sum_{i=1}^{N}a_{i}=M$. Note that the feasible actions are independent of the state and the time.
- $P_{N}$ denotes the state transition probabilities. We define $P_{x,x^{\prime }}\left(a\right)$ as the probability that action a at state x will lead to state $x^{\prime }$. It is calculated by
  $$P_{x,x^{\prime }}\left(a\right)=\prod_{i=1}^{N}P\left({\hat{r}}_{i}^{\prime }\right)P_{s_{i},s_{i}^{\prime }}\left(a_{i},{\hat{r}}_{i}\right),$$
  where $P_{s_{i},s_{i}^{\prime }}\left(a_{i},{\hat{r}}_{i}\right)$ is the transition probability from $s_{i}$ to $s_{i}^{\prime }$ when the estimate of CSI is ${\hat{r}}_{i}$ and action $a_{i}$ is taken. The values of $P_{s_{i},s_{i}^{\prime }}\left(a_{i},{\hat{r}}_{i}\right)$ can be obtained easily from the results in Section 2.3.
- $C_{N}\left(w\right)$ denotes the instant cost. When the system is at state x and action a is taken, the instant cost is $C\left(x,a\right)≜\sum_{i=1}^{N}C\left(x_{i},a_{i}\right)≜\sum_{i=1}^{N}\left(f_{i}\left(s_{i}\right)+wa_{i}\right)$.

We notice that PP can be cast into $M_{N}\left(0,M\right)$. Since w=0, the instant cost is independent of action a. Therefore, we abbreviate C(x,a) as C(x). To simplify the analysis, we consider the case of M=1. Equivalently, we investigate the structural properties of the optimal policy for $M_{N}\left(0,1\right)$.

**Remark** **2.** *For the case of M>1, we can apply the same methodology. However, as M increases, the action space will grow quickly, resulting in the need to consider more feasible actions in each step of the proof. Hence, to better demonstrate the methodology, we only consider the case of M=1 in this paper.*

It is well known that the optimal policy for $M_{N}\left(0,1\right)$ can be characterized by the value function. We denote the value function of state x as V(x). A canonical procedure to calculate V(x) is applying the Value Iteration Algorithm (VIA). To this end, we define $V_{\nu }\left(\cdot \right)$ as the estimated value function at iteration ν of VIA and initialize $V_{0}\left(\cdot \right)=0$. Then, VIA updates the estimated value functions in the following way

(5) $$\begin{array}{cc} V_{\nu +1}\left(x\right) & =C\left(x\right)-\theta +\min_{a\in A_{N}\left(1\right)}\left\{\sum_{x^{\prime }\in X_{N}}P_{x,x^{\prime }}\left(a\right)V_{\nu }\left(x^{\prime }\right)\right\}, \end{array}$$

where θ is the optimal value of $M_{N}\left(0,1\right)$. VIA is guaranteed to converge to the value function [21]. More precisely, $V_{\nu }\left(\cdot \right)=V\left(\cdot \right)$ when ν→+∞. However, the exact value function is impossible to get since we need infinite iterations and the state space is infinite. Instead, we provide two structural properties of the value function.

**Lemma** **1** (Monotonicity)**.** *For $M_{N}\left(0,1\right)$, V(x) is non-decreasing in $s_{i}$ for 1≤i≤N.*

**Proof.**  Leveraging the iterative nature of VIA, we use mathematical induction to prove the desired results. The complete proof can be found in Appendix A.    ☐

Before introducing the next structural property, we make the following definition.

**Definition** **1** (Statistically identical)**.** *Two users are said to be statistically identical if the user-dependent parameters and the adopted time penalty functions are the same.*

For the users that are statistically identical, we can prove the following

**Lemma** **2** (Equivalence)**.** *For $M_{N}\left(0,1\right)$, if users j and k are statistically identical, V(x)=V(P(x)) where P(x) is state x with $x_{j}$ and $x_{k}$ exchanged.*

**Proof.**  Leveraging the iterative nature of VIA, we use mathematical induction to prove the desired results. At each iteration, we show that for each feasible action at state x, we can find an equivalent action at state P(x). Two actions are equivalent if they lead to the same value function. The complete proof can be found in Appendix B.    ☐

Equipped with the above lemmas, we proceed with characterizing the structural properties of the optimal policy. We recall that the optimal action at each state can be characterized by the value function. Hence, we denote, by $V^{j}\left(x\right)$, the value function resulting from choosing user *j* to update at state x. Then, $V^{j}\left(x\right)$ can be calculated by

$$V^{j}\left(x\right)=C\left(x\right)-\theta +\sum_{x^{\prime }-x_{j}^{\prime }}\left\{\left(\prod_{i\neq j}P_{x_{i},x_{i}^{\prime }}\left(0\right)\right)\sum_{{\hat{r}}_{j}^{\prime }}\left[P\left({\hat{r}}_{j}^{\prime }\right)\left(\sum_{s_{j}^{\prime }}P_{s_{j},s_{j}^{\prime }}\left(1,{\hat{r}}_{j}\right)V\left(x^{\prime }\right)\right)\right]\right\}.$$

If $V^{j}\left(x\right)<V^{k}\left(x\right)$ for all k≠j, it is optimal to transmit the update from user *j*. When $V^{j}\left(x\right)=V^{k}\left(x\right)$, the two choices are equally desirable. In the following, we will characterize the properties of $\delta^{j,k}\left(x\right)≜V^{j}\left(x\right)-V^{k}\left(x\right)$ for any *j* and *k*.

**Theorem** **1** (Structural properties)**.** *For $M_{N}\left(0,1\right)$, $\delta^{j,k}\left(x\right)$ has the following properties*

1. *$\delta^{j,k}\left(x\right)\leq 0$ if ${\hat{r}}_{k}=p_{e,k}^{0}=0$. The equality holds when $s_{j}=0$ or ${\hat{r}}_{j}=p_{e,j}^{0}=0$.*
2. *$\delta^{j,k}\left(x\right)$ is non-increasing in ${\hat{r}}_{j}$ and is non-decreasing in ${\hat{r}}_{k}$ when $s_{j},s_{k}>0$. At the same time, $\delta^{j,k}\left(x\right)$ is independent of ${\hat{r}}_{i}$ for any i≠j,k.*
3. *$\delta^{j,k}\left(x\right)\leq 0$ if $s_{k}=0$. The equality holds when $s_{j}=0$ or ${\hat{r}}_{j}=p_{e,j}^{0}=0$.*
4. *$\delta^{j,k}\left(x\right)$ is non-increasing in $s_{j}$ if $\Gamma_{j}^{{\hat{r}}_{j}}\leq \Gamma_{k}^{{\hat{r}}_{k}}$ and is non-decreasing in $s_{k}$ if $\Gamma_{j}^{{\hat{r}}_{j}}\geq \Gamma_{k}^{{\hat{r}}_{k}}$ when $s_{j},s_{k}>0$. We define $\Gamma_{i}^{1}≜\frac{\alpha_{i}}{1-p_{i}}$ and $\Gamma_{i}^{0}≜\frac{\beta_{i}}{1-p_{i}}$ for 1≤i≤N.*
5. *$\delta^{j,k}\left(x\right)\leq 0$ if $s_{j}\geq s_{k}$, ${\hat{r}}_{j}\geq {\hat{r}}_{k}$, and users j and k are statistically identical.*

**Proof.**  The proof can be found in Appendix C.    ☐

We notice that $\Gamma_{i}^{{\hat{r}}_{i}}$ can be written as

$$\Gamma_{i}^{{\hat{r}}_{i}}=\frac{Pr(s_{i}+1|\left(s_{i},{\hat{r}}_{i}\right),a_{i}=1)}{Pr(s_{i}+1|\left(s_{i},{\hat{r}}_{i}\right),a_{i}=0)}<1,$$

where $s_{i}$ can be any positive integer. Consequently, $\Gamma_{i}^{{\hat{r}}_{i}}$ is independent of any $s_{i}>0$ and indicates the decrease in the probability of increasing $s_{i}$ caused by action $a_{i}=1$. When $\Gamma_{i}^{{\hat{r}}_{i}}$ is large, action $a_{i}=1$ will achieve a small decrease in the probability of increasing $s_{i}$. In the following, we provide an intuitive interpretation of why the monotonicity in Property 4 of Theorem 1 depends on $\Gamma_{i}^{{\hat{r}}_{i}}$. We take the case of $\Gamma_{j}^{{\hat{r}}_{j}}\leq \Gamma_{k}^{{\hat{r}}_{k}}$ as an example and assume that there are only users *j* and *k* in the system. Then, according to Section 2.3, the dynamic of $s_{j}$ and $s_{k}$ can be divided into the following three cases

- Neither $s_{j}$ nor $s_{k}$ increases. In this case, both $s_{j}$ and $s_{k}$ become zero.
- Either $s_{j}$ or $s_{k}$ increases and the other becomes zero. We denote by $P_{j}^{k}$ the probability that only $s_{k}$ increases when $a_{j}=1$. The notation for other cases is defined analogously. The probabilities can be obtained easily using the results in Section 2.3.
- Both $s_{j}$ and $s_{k}$ increase. We denote by $P_{j}$ the probability that both $s_{j}$ and $s_{k}$ increase when $a_{j}=1$. $P_{k}$ is defined analogously. The probabilities can be obtained easily using the results in Section 2.3.

We notice that $\delta^{j,k}\left(x\right)$ implies the tendency of the base station to choose between the two users. The larger $\delta^{j,k}\left(x\right)$ is, the more the base station tends to choose user *k*. Thus, we investigate the base station’s propensity to choose user *k* when $s_{k}$ increases but $s_{j}$ stays the same. We ignore the case where the resulting $s_{k}$ is zero since it is independent of the increase in $s_{k}$. With this in mind, we first notice that $P_{k}^{k}\leq P_{j}^{k}$. Meanwhile, we can easily verify that $\frac{P_{j}}{P_{k}}=\frac{\Gamma_{j}^{{\hat{r}}_{j}}}{\Gamma_{k}^{{\hat{r}}_{k}}}$. When $\Gamma_{j}^{{\hat{r}}_{j}}\leq \Gamma_{k}^{{\hat{r}}_{k}}$, we have $P_{j}\leq P_{k}$. Then, there exists a subtle trade-off. More precisely, choosing user *k* will result in $P_{k}^{k}\leq P_{j}^{k}$, but at the cost of $P_{k}\geq P_{j}$. Hence, in this case, the propensity of the base station is hard to determine. Following the same line, we can show that choosing user *j* will lead to $P_{j}^{j}\leq P_{k}^{j}$ and $P_{j}\leq P_{k}$. Thus, there exists no such trade-off when we investigate the base station’s propensity to choose user *j* as $s_{j}$ increases but $s_{k}$ stays the same.

Leveraging Theorem 1, we can provide some specific structural properties of the optimal policy.

**Corollary** **1** (Application of Theorem 1)**.** *When M=1, the optimal policy for PP must satisfy the following*

1. *The user i with ${\hat{r}}_{i}=p_{e,i}^{0}=0$ or $s_{i}=0$ will not be chosen unless it is to break the tie.*
2. *When user j is chosen at state $x_{1}$, then for state $x_{2}$, such that ${\hat{r}}_{1,j}\leq {\hat{r}}_{2,j}$ and $s_{1,i}=s_{2,i}$ for 1≤i≤N, the optimal choice must be in the set $G=\left\{j\right\}\cup \left\{k:{\hat{r}}_{1,k}<{\hat{r}}_{2,k}\right\}$.*
3. *When N=2, we consider two states, $x_{1}$ and $x_{2}$, which differ only in the value of $s_{j}$. Specifically, $s_{1,j}\leq s_{2,j}$. If user j is chosen at state $x_{1}$ and $\Gamma_{j}^{{\hat{r}}_{1,j}}\leq \Gamma_{k}^{{\hat{r}}_{1,k}}$, the optimal choice at state $x_{2}$ will also be user j.*
4. *When N=2, we consider two states, $x_{1}$ and $x_{2}$, which differ only in the value of $s_{k}$. Specifically, $s_{1,k}\geq s_{2,k}$. If user j is chosen at state $x_{1}$ and $\Gamma_{j}^{{\hat{r}}_{1,j}}\geq \Gamma_{k}^{{\hat{r}}_{1,k}}$, the optimal choice at state $x_{2}$ will also be user j.*
5. *When all users are statistically identical, the optimal choice at any time slot must be either the user with $x=(s_{max,1},1)$ where $s_{max,1}≜\max_{s_{i}}\left\{\left(s_{i},1\right)\right\}$ or the user with $x=(s_{max,0},0)$ where $s_{max,0}≜\max_{s_{i}}\left\{\left(s_{i},0\right)\right\}$. Moreover,*
  - *If $s_{max,1}\geq s_{max,0}$, it is optimal to choose the user with $x=(s_{max,1},1)$.*
  - *If $s_{max,1}<s_{max,0}$, the optimal choice will switch from the user with $x=(s_{max,0},0)$ to the user with $x=(s_{max,1},1)$ when $s_{max,1}$ increases from 0 to $s_{max,0}$ solely.*

**Proof.**  The first property follows directly from Property 1 and Property 3 of Theorem 1. For the second property, leveraging Property 2 of Theorem 1, we have $\delta^{j,k}\left(x_{2}\right)\leq \delta^{j,k}\left(x_{1}\right)\leq 0$ if ${\hat{r}}_{1,j}\leq {\hat{r}}_{2,j}$, ${\hat{r}}_{1,k}\geq {\hat{r}}_{2,k}$, and $s_{1,i}=s_{2,i}$ for 1≤i≤N. Thus, the optimal choice will not be user *k* in this case. Then, we can conclude that the optimal choice must be in the set $G=\left\{j\right\}\cup \left\{k:{\hat{r}}_{1,k}<{\hat{r}}_{2,k}\right\}$.

For the third property, we have proved in Property 4 of Theorem 1 that $\delta^{j,k}\left(x\right)$ is non-increasing in $s_{j}$ if $\Gamma_{j}^{{\hat{r}}_{j}}\leq \Gamma_{k}^{{\hat{r}}_{k}}$. Hence, $\delta^{j,k}\left(x_{2}\right)\leq \delta^{j,k}\left(x_{1}\right)\leq 0$. As we consider the case of N=2, the optimal choice at state $x_{2}$ will also be user *j*. The fourth property can be shown in a similar way by noticing that $\delta^{j,k}\left(x\right)$ is non-decreasing in $s_{k}$ when $\Gamma_{j}^{{\hat{r}}_{j}}\geq \Gamma_{k}^{{\hat{r}}_{k}}$.

For the last property, we recall from Property 5 of Theorem 1 that it is always better to choose the user with a larger *s* if they are statistically identical and have the same $\hat{r}$. Thus, we can conclude that the optimal choice must be either the user with $x=(s_{max,1},1)$ or the user with $x=(s_{max,0},0)$. Without a loss of generality, we assume $x_{j}=\left(s_{max,1},1\right)$ and $x_{k}=\left(s_{max,0},0\right)$. Now, we distinguish between the following cases

- According to Property 5 of Theorem 1, we can conclude that it is optimal to choose user *j* when $s_{max,1}\geq s_{max,0}$.
- To determine the optimal choice in the case of $s_{max,1}<s_{max,0}$, we recall that the optimal choice will be user *k* (i.e., $\delta^{j,k}\left(x\right)\geq 0$) if $s_{j}=0$ and will be user *j* (i.e., $\delta^{j,k}\left(x\right)\leq 0$) if $s_{j}=s_{k}$. At the same time, Property 4 of Theorem 1 tells us that $\delta^{j,k}\left(x\right)$ is non-increasing in $s_{j}$ when users *j* and *k* are statistically identical. Therefore, we can conclude that the optimal choice will switch from user *k* to user *j* when $s_{j}$ increases from 0 to $s_{k}$ solely.

   ☐

## 4. Whittle’s Index Policy

Whittle’s index policy is a well-known low-complexity heuristic that shows a strong performance in many problems that belong to RMAB [22,23,24]. In this section, we develop Whittle’s index policy for PP. We first present the general procedures we adopt to obtain Whittle’s index.

- We first formulate a relaxed version of PP and apply the Lagrangian approach.
- Then, we decouple the problem of minimizing the Lagrangian function into N decoupled problems, each of which only considers a single user. By casting the decoupled problem into an MDP, we investigate the structural properties and performance of the optimal policy.
- Leveraging the results above and under a simple condition, we establish the indexability of the decoupled problem.
- Finally, we obtain the expression of Whittle’s index by solving the Bellman equation.

### 4.1. Relaxed Problem

The first step in obtaining Whittle’s index is to formulate the Relaxed Problem (RP). More precisely, instead of requiring the limit on the number of updates allowed per transmission attempt to be met in each time slot, we relax the constraint such that the limit is not violated in an average sense. Then, RP can be formulated as

(6a) $$\begin{array}{cc} \underset{\phi \;\in \;\Phi }{\arg\;\min} & \bar{\Delta }\;≜\;\lim_{T\to \infty }\frac{1}{T}E_{\phi }(\sum_{t=0}^{T-1}\sum_{i=1}^{N}f_{i}(s_{i,t})) \end{array}$$

(6b) $$\begin{array}{cc} \mathrm{subject}\;\mathrm{to} & {\bar{\rho }}_{\phi }\;≜\;\lim_{T\to \infty }\frac{1}{T}E_{\phi }(\sum_{t=0}^{T-1}\sum_{i=1}^{N}a_{i,t})\;\leq \;M. \end{array}$$

As RP is specified, we apply the Lagrangian approach. First of all, we write RP into its Lagrangian form.

$$L\left(\lambda ,\phi \right)=\lim_{T\to \infty }\frac{1}{T}E_{\phi }\left(\sum_{t=0}^{T-1}\sum_{i=1}^{N}\left(f_{i}\left(s_{i,t}\right)+\lambda a_{i,t}\right)\right)-\lambda M,$$

where λ≥0 is the Lagrange multiplier. Then, we investigate the problem of minimizing the Lagrangian function. Since λM is independent of policies, we can ignore it. More precisely, we consider the following minimization problem

(7) $$\begin{array}{cc} \underset{\phi \;\in \;\Phi }{\mathrm{minimize}} & \lim_{T\to \infty }\frac{1}{T}E_{\phi }(\sum_{t=0}^{T-1}\sum_{i=1}^{N}(f_{i}(s_{i,t})\;+\;\lambda a_{i,t}). \end{array}$$

### 4.2. Decoupled Model

In this section, we formulate the decoupled problem and investigate its optimal policy. The decoupled model associated with each user follows the system model with N=1. Since all the users share the same structure, we drop the user-dependent subscript *i* for simplicity. Then, the decoupled problem can be formulated as

(8) $$\begin{array}{cc} \underset{\phi \;\in \;\Phi^{\prime }}{\mathrm{minimize}} & \lim_{T\to \infty }\frac{1}{T}E_{\phi }(\sum_{t=0}^{T-1}(f(s_{t})\;+\;\lambda a_{t})), \end{array}$$

where $\Phi^{\prime }$ is the set of all causal policies when N=1. We notice that problem (8) can be cast into the MDP $M_{1}\left(\lambda ,-1\right)$. We define M=−1 when there is no restriction on the number of updates allowed per transmission attempt.

We first investigate the structural properties of the optimal policy for $M_{1}\left(\lambda ,-1\right)$ when λ is a given non-negative constant. We start with characterizing the corresponding value function V(x).

**Corollary** **2** (Extension of Lemma 1)**.** *For $M_{1}\left(\lambda ,-1\right)$, V(x) is non-decreasing in s.*

**Proof.**  The proof follows the same steps as in the proof of Lemma 1. The complete proof can be found in Appendix D.    ☐

Equipped with the above corollary, we can characterize the structural properties of the optimal policy for (8).

**Proposition** **1** (Optimal policy for decoupled problem)**.** *The optimal policy for the decoupled problem is a threshold policy with the following properties.*

- *The optimal policy can be fully captured by $n=(n_{0},n_{1})$. More precisely, when the system is at state $\left(s,\hat{r}\right)$, it is optimal to make a transmission attempt only when $s\geq n_{\hat{r}}$.*
- *$n_{0}\geq n_{1}>0$.*

**Proof.**  We define $\Delta V\left(x\right)≜V^{1}\left(x\right)-V^{0}\left(x\right)$, where $V^{a}\left(x\right)$ is the value function resulting from taking action *a* at state *x*. Then, the optimal action at state *x* is a=1 if ΔV(x)<0, and a=0 is optimal otherwise. We use Corollary 2 to characterize the sign of ΔV(x). The complete proof can be found in Appendix E.    ☐

In the following, we evaluate the performance of the threshold policy detailed in Proposition 1. More precisely, we calculate the expected AoII ${\bar{\Delta }}_{n}$ and the expected transmission rate ${\bar{\rho }}_{n}$ resulting from the adoption of threshold policy n. We will see in the following that ${\bar{\Delta }}_{n}$ and ${\bar{\rho }}_{n}$ are essential for establishing the indexability and obtaining the expression of Whittle’s index.

**Proposition** **2** (Performance)**.** *Under threshold policy $n=(n_{0},n_{1})$,*

$${\bar{\Delta }}_{n}=\pi_{0}p\left[\sum_{k=1}^{n_{1}-1}f\left(k\right){\left(1-p\right)}^{k-1}+{\left(1-p\right)}^{n_{1}-1}\left(\sum_{k=n_{1}}^{n_{0}-1}f\left(k\right)c_{1}^{k-n_{1}}+c_{1}^{n_{0}-n_{1}}\sum_{k=n_{0}}^{+\infty }f\left(k\right)c_{2}^{k-n_{0}}\right)\right],$$

$${\bar{\rho }}_{n}=\pi_{0}p{\left(1-p\right)}^{n_{1}-1}\left[\frac{\gamma }{1-c_{1}}+c_{1}^{n_{0}-n_{1}}\left(\frac{1}{1-c_{2}}-\frac{\gamma }{1-c_{1}}\right)\right],$$

*where*

$$\pi_{0}=\frac{1}{2+p{\left(1-p\right)}^{n_{1}-1}\left[\frac{1}{1-c_{1}}-\frac{1}{p}+c_{1}^{n_{0}-n_{1}}\left(\frac{1}{1-c_{2}}-\frac{1}{1-c_{1}}\right)\right]},$$

*$c_{1}=\left(1-\gamma \right)\left(1-p\right)+\gamma \alpha$, and $c_{2}=\left(1-\gamma \right)\beta +\gamma \alpha$.*

**Proof.**  We notice that the dynamic of AoII under the threshold policy can be fully captured by a Discrete-Time Markov Chain (DTMC). Then, combined with the fact that $\hat{r}$ is an independent Bernoulli random variable, we can obtain the desired results from the stationary distribution of the induced DTMC. The complete proof can be found in Appendix F.    ☐

As f(·) can be any non-decreasing function, $\bar{\Delta }$ can grow indefinitely. Thus, it is necessary to require that there exists at least one threshold policy that causes a finite $\bar{\Delta }$. By noting that $1-p\geq c_{1}\geq c_{2}$, we have

$$\begin{array}{cc} \bar{\Delta } & \geq \pi_{0}p\left[\sum_{k=1}^{n_{1}-1}f\left(k\right)c_{2}^{k-1}+c_{2}^{n_{1}-1}\left(\sum_{k=n_{1}}^{n_{0}-1}f\left(k\right)c_{2}^{k-n_{1}}+c_{2}^{n_{0}-n_{1}}\sum_{k=n_{0}}^{+\infty }f\left(k\right)c_{2}^{k-n_{0}}\right)\right]\\ \; & =\pi_{0}p\left(\sum_{k=1}^{+\infty }f\left(k\right)c_{2}^{k-1}\right). \end{array}$$

The equality is achieved when $n_{0}=n_{1}=1$. Then, we can conclude that it is sufficient to require $\sum_{k=1}^{+\infty }f\left(k\right)c_{2}^{k-1}<+\infty$. This will be the underlying assumption throughout the rest of this paper.

### 4.3. Indexability

In this section, we establish the indexability of the decoupled problem, which ensures the existence of Whittle’s index. We start with the definition of indexability.

**Definition** **2** (Indexability). *The decoupled problem is indexable if the set of states in which a=0 is the optimal action increases with λ, that is,*

$$\lambda^{\prime }<\lambda ⟹D\left(\lambda^{\prime }\right)\subseteq D\left(\lambda \right),$$

*where D(λ) is the set of states in which a=0 is optimal when Lagrange multiplier λ is adopted.*

The Lagrange multiplier λ can be viewed as a cost associated with each transmission attempt. Intuitively, as λ increases, the base station should stay idle (i.e., a=0) for a longer time until *s* becomes large enough to offset the cost. Although it is intuitively correct that the decoupled problem is indexable, the indexability is hard to establish as the optimal policy is characterized by two thresholds. Thus, Whittle’s index does not necessarily exist. However, the indexability can be established when the following condition is satisfied

(9) $$p_{e,i}^{0}=0\;for\;1\leq i\leq N.$$

**Remark** **3.** *Problem *(9)* only requires the estimate ${\hat{r}}_{i}$ to be perfect when ${\hat{r}}_{i}=0$. In the case of ${\hat{r}}_{i}=1$, we still allow the estimate to be inaccurate.*

When (9) is satisfied, Propositions 1 and 2 reduce to the following

**Corollary** **3** (Consequences of (9))**.** *When *(9)* is satisfied, the optimal policy for the decoupled problem *(8)* is the threshold policy n=(+∞,n). The corresponding ${\bar{\Delta }}_{n}$ and ${\bar{\rho }}_{n}$ are*

$${\bar{\Delta }}_{n}=\pi_{0}p\left(\sum_{k=1}^{n-1}f\left(k\right){\left(1-p\right)}^{k-1}+{\left(1-p\right)}^{n-1}\sum_{k=n}^{+\infty }f\left(k\right)c_{1}^{k-n}\right),$$

$${\bar{\rho }}_{n}=\pi_{0}p{\left(1-p\right)}^{n-1}\left(\frac{\gamma }{1-c_{1}}\right),$$

*where*

$$\pi_{0}=\frac{1}{2+p{\left(1-p\right)}^{n-1}\left(\frac{1}{1-c_{1}}-\frac{1}{p}\right)}.$$

**Proof.**  We continue with the same notations as in the proof of Propositions 1 and 2. It is sufficient to show that $n_{0}=+\infty$. To this end, we consider the state x=(s,0). By following the same steps as in the proof of Proposition 1, we have

ΔV(s,0)=λ≥0.

Therefore, it is optimal to stay idle (i.e., a=0) at state x=(s,0) for any s≥0. Equivalently, $n_{0}=+\infty$. Then, the corresponding ${\bar{\Delta }}_{n}$ and ${\bar{\rho }}_{n}$ can be calculated as a special case of Proposition 2 where $n_{0}=+\infty$, $n_{1}=n$, and $p_{e}^{0}=0$.    ☐

Leveraging Corollary 3, we can establish the indexability of the decoupled problem.

**Proposition** **3** (Indexability of decoupled problem)**.** *The decoupled problem is indexable when (9) is satisfied.*

**Proof.**  According to Proposition 2.2 of [25], we only need to verify that the expected transmission rate ${\bar{\rho }}_{n}$ is strictly decreasing in *n*. From Corollary 3, we have

$$\begin{array}{c} {\bar{\rho }}_{n}=\frac{\gamma \left(\frac{p}{1-c_{1}}\right)}{\frac{2}{{\left(1-p\right)}^{n-1}}+\left(\frac{p}{1-c_{1}}-1\right)}. \end{array}$$

As $\frac{1}{2}<1-p<1$, we can easily verify that ${\bar{\rho }}_{n}$ is strictly decreasing in *n*. Thus, the decoupled problem is indexable when (9) is satisfied.    ☐

### 4.4. Whittle’s Index Policy

In this section, we proceed with finding the expression of Whittle’s index and defining Whittle’s index policy. First of all, we give the definition of Whittle’s index.

**Definition** **3** (Whittle’s index)**.** *When the decoupled problem is indexable, Whittle’s index at state x is defined as the infimum λ, such that both actions are equally desirable. Equivalently, Whittle’s index at state x is defined as the infimum λ such that $V^{0}\left(x\right)=V^{1}\left(x\right)$.*

Let us denote by $W_{x}$ the Whittle’s index at state *x*. Then, the expression of Whittle’s index is given by the following Proposition.

**Proposition** **4** (Whittle’s index)**.** *When (9) is satisfied, Whittle’s index is*

$$W_{x}=\left\{\begin{array}{cc} 0 & when\;x=\left(0,\hat{r}\right)\;or\;x=\left(s,0\right),\\ \frac{\left(1-c_{1}\right)\sum_{k=s+1}^{+\infty }f\left(k\right)c_{1}^{k-s-1}-{\bar{\Delta }}_{s}}{\frac{\left(1-c_{1}\right)\left(1-p\right)-\gamma \left(1-p-\alpha \right)}{c_{1}\left(1-p-\alpha \right)}+{\bar{\rho }}_{s}} & when\;x=(s,1), \end{array}\right.$$

*where s>0 and $c_{1}=\left(1-\gamma \right)\left(1-p\right)+\gamma \alpha$. ${\bar{\Delta }}_{s}$ and ${\bar{\rho }}_{s}$ are the expected AoII and the expected transmission rate when threshold policy n=(+∞,s) is adopted, respectively. At the same time, $W_{x}$ is non-negative and is non-decreasing in s.*

**Proof.**  Whittle’s indexes at state $x=(0,\hat{r})$ and x=(s,0) are obtained easily from the proof of Proposition 1. For state x=(s,1), we first use backward induction to calculate the expressions of some value functions. Then, the expression of Whittle’s index can be obtained from its definition. The complete proof can be found in Appendix G.    ☐

**Definition** **4** (Whittle’s index policy). *At any state $x=(x_{1},x_{2},\ldots ,x_{N})$, the base station will transmit the updates from M users with the largest $W_{x_{i}}$. The ties are broken arbitrarily. $W_{x_{i}}$ is calculated using Proposition 4 with the parameters of user i.*

**Remark** **4.** *Whittle’s index policy possesses the structural properties detailed in Corollary 1.*

- *The first two properties can be verified by noting that $W_{x_{i}}\geq 0$ and the equality holds when ${\hat{r}}_{i}=0$ or $s_{i}=0$. At the same time, $W_{x_{i}}$ is non-decreasing in ${\hat{r}}_{i}$.*
- *The third and fourth properties can be verified by noting that $W_{x_{i}}$ is non-decreasing in $s_{i}$.*
- *For the last property, we first notice that $W_{x_{j}}=W_{x_{k}}$ when users j and k are statistically identical and $x_{j}=x_{k}$. Then, the property can be verified by noting that $W_{x_{i}}$ is non-decreasing in both $s_{i}$ and ${\hat{r}}_{i}$.*

## 5. Optimal Policy for Relaxed Problem

In this section, we provide an efficient algorithm to obtain the optimal policy for RP, based on which we will develop another scheduling policy for PP in the next section that is free from indexability. At the same time, the performance of the optimal policy for RP forms a universal lower bound because the following ordering holds

$${\bar{\Delta }}_{AoII}^{RP}\leq {\bar{\Delta }}_{AoII}^{PP},$$

where ${\bar{\Delta }}_{AoII}^{RP}$ and ${\bar{\Delta }}_{AoII}^{PP}$ are the minimal expected AoII of RP and PP, respectively.

**Remark** **5.** *Note that the optimal policy for RP may not necessarily be a valid policy for PP, as the transmitter may transmit more than M updates in one transmission attempt under RP-optimal policy.*

To solve RP, we follow the discussion in Section 4.1. More precisely, we take the Lagrangian approach and consider the problem reported in (7). We will see in the following discussion that the optimal policy for RP can be characterized by the optimal policies for problem (7). Therefore, we first cast problem (7) into the MDP $M_{N}\left(\lambda ,-1\right)$. However, the optimal policy for $M_{N}\left(\lambda ,-1\right)$ is difficult to obtain because the state space is infinite. Even though we can make the state space finite by imposing an upper limit on the value of *s*, the state space and the action space grow exponentially with the number of users in the system. To overcome the difficulty, we investigate the optimal policy for $M_{1}^{i}\left(\lambda ,-1\right)$ where 1≤i≤N. The superscript *i* means that the only user in the system is user *i*. We will show later that the optimal policy for $M_{N}\left(\lambda ,-1\right)$ can be fully characterized by the optimal policies for $M_{1}^{i}\left(\lambda ,-1\right)$ where 1≤i≤N.

### 5.1. Optimal Policy for Single User

In this section, we tackle the problem of finding the optimal policy for $M_{1}^{i}\left(\lambda ,-1\right)$. Since the users share the same structure, we ignore the superscript *i* for simplicity. To find the optimal policy, we first use the Approximating Sequence Method (ASM) introduced in [26] to make the state space finite. More precisely, we impose s≤m where *m* is a predetermined upper limit. The state transition probabilities $P_{s,s^{\prime }}^{\prime }\left(a,\hat{r}\right)$ are modified in the following way

(10) $$P_{s,s^{\prime }}^{\prime }\left(a,\hat{r}\right)=\left\{\begin{array}{cc} P_{s,s^{\prime }}\left(a,\hat{r}\right) & if\;\;s^{\prime }<m,\\ P_{s,s^{\prime }}\left(a,\hat{r}\right)+\sum_{z>m}P_{s,z}\left(a,\hat{r}\right) & if\;\;s^{\prime }=m. \end{array}\right.$$

The action space and the instant cost remain unchanged. Then, we can apply Relative Value Iteration (RVI) with convergence criteria ϵ to obtain the optimal policy. We notice that $M_{1}\left(\lambda ,-1\right)$ coincides with the decoupled model studied in Section 4.2. Hence, we can utilize the threshold structure of the optimal policy to improve RVI. To this end, we class a state as active if the optimal action at this state is a=1. Then, the threshold structure detailed in Proposition 1 tells us the following. For any state *x*, if there exists an active state $x_{1}$ with $s_{1}\leq s$ and ${\hat{r}}_{1}\leq \hat{r}$, then *x* must also be active. Hence, we can determine the optimal action at state *x* immediately instead of comparing all feasible actions. In this way, we can reduce the running time of RVI. The pseudocode for the improved RVI can be found in Algorithm A1 of Appendix M. A similar technique is also presented in [5].

For $M_{1}\left(\lambda ,-1\right)$, when problem (9) is satisfied, Whittle’s index exists and can be calculated efficiently using Proposition 4. Therefore, we can obtain the optimal policy using Whittle’s index and further reduce the computational complexity. To this end, we denote by $n_{\lambda }$ the optimal policy for $M_{1}\left(\lambda ,-1\right)$ and present the following proposition

**Proposition** **5** (Optimal deterministic policy)**.** *When *(9)* is satisfied, the optimal policy for $M_{1}\left(\lambda ,-1\right)$ is $n_{\lambda }=\left(+\infty ,n\right)$ where n is given by*

$$n=\left\{\begin{array}{cc} 1 & if\;\lambda =0,\\ \max\{s\in N_{0}:W_{s}\leq \lambda \}+1 & if\;\lambda >0. \end{array}\right.$$

*$W_{s}$ is the Whittle’s index at state (s,1).*

**Proof.**  We first notice that $M_{1}\left(\lambda ,-1\right)$ coincides with the decoupled model studied in Section 4.2. Then, we show the optimal action for each state with $\hat{r}=1$ using the definition of Whittle’s index and the fact that the decoupled problem is indexable when (9) is satisfied. The complete proof can be found in Appendix H.    ☐

In the following, we provide a randomized policy that is also optimal for $M_{1}\left(\lambda ,-1\right)$. We will see later that the randomized policy is the key to obtaining the optimal policy for RP.

**Theorem** **2** (Optimal randomized policy)**.** *There exist two deterministic policies $n_{\lambda_{+}}$ and $n_{\lambda_{-}}$, which are both optimal for $M_{1}\left(\lambda ,-1\right)$. We consider the following randomized policy $n_{\lambda }$: every time the system reaches state (0,0), the base station will make the choice between $n_{\lambda_{-}}$ with probability μ and $n_{\lambda_{+}}$ with probability 1−μ. The chosen policy will be followed until the next choice. Then, the randomized policy $n_{\lambda }$ is optimal for $M_{1}\left(\lambda ,-1\right)$ under any μ∈[0,1].*

**Proof.**  We show that our system verifies the assumptions given in [27]. Then, leveraging the characteristics of our system, we can obtain the optimal randomized policy. The complete proof can be found in Appendix I.    ☐

In practice, we approximate $\lambda_{+}\approx \lambda +\xi$ and $\lambda_{-}\approx \lambda -\xi$ where ξ is a small perturbation. Then, the deterministic policies $n_{\lambda_{+}}$ and $n_{\lambda_{-}}$ can be obtained by following the discussion at the beginning of this subsection. Note that, in most cases, $n_{\lambda_{+}}$ and $n_{\lambda_{-}}$ are the same.

### 5.2. Optimal Policy for RP

In this section, we characterize the optimal policy for RP. Let us denote by V(x) and $V^{i}\left(x_{i}\right)$ the value functions of $M_{N}\left(\lambda ,-1\right)$ and $M_{1}^{i}\left(\lambda ,-1\right)$, respectively. Then, we can prove the following

**Proposition** **6** (Separability)**.** *$V\left(x\right)=\sum_{i=1}^{N}V^{i}\left(x_{i}\right)$ where $x=(x_{1},\ldots ,x_{N})$. In other words, the policy, under which each user adopts its own optimal policy, is optimal for $M_{N}\left(\lambda ,-1\right)$.*

**Proof.**  We show $V\left(x\right)=\sum_{i=1}^{N}V^{i}\left(x_{i}\right)$ by comparing the Bellman equations they must satisfy. The complete proof can be found in Appendix J.    ☐

We denote the optimal policy for $M_{N}\left(\lambda ,-1\right)$ as $\phi_{\lambda }=\left[n_{\lambda ,1},\ldots ,n_{\lambda ,N}\right]$ where $n_{\lambda ,i}$ is the optimal policy for $M_{1}^{i}\left(\lambda ,-1\right)$. For simplicity, we define $\bar{\Delta }\left(\lambda \right)$ and $\bar{\rho }\left(\lambda \right)$ as the expected AoII and the expected transmission rate associated with $\phi_{\lambda }$, respectively. ${\bar{\Delta }}^{i}\left(\lambda \right)$ and ${\bar{\rho }}^{i}\left(\lambda \right)$ are defined analogously for user *i* under policy $n_{\lambda ,i}$. We also define $\lambda^{*}≜\inf\{\lambda >0:$$\bar{\rho }\left(\lambda \right)\leq M\}$. With Proposition 6 and the above definitions in mind, we proceed with constructing the optimal policy for RP.

**Theorem** **3** (Optimal policy for RP)**.** The optimal policy for RP can be characterized by two deterministic policies $\phi_{\lambda_{+}^{*}}=\left[n_{\lambda_{+}^{*},1},\ldots ,n_{\lambda_{+}^{*},N}\right]$ and $\phi_{\lambda_{-}^{*}}=\left[n_{\lambda_{-}^{*},1},\ldots ,n_{\lambda_{-}^{*},N}\right]$ where $n_{\lambda_{+}^{*},i}$ and $n_{\lambda_{-}^{*},i}$ are both the optimal deterministic policies for $M_{1}^{i}\left(\lambda^{*},-1\right)$. Then, we mix $\phi_{\lambda_{+}^{*}}$ and $\phi_{\lambda_{-}^{*}}$ in the following way: for each user i, every time the user reaches state (0,0), the base station will make the choice between $n_{\lambda_{-}^{*},i}$ with probability $\mu_{i}$ and $n_{\lambda_{+}^{*},i}$ with probability $1-\mu_{i}$. The chosen policy will be followed by user i until the next choice. Where 1≤i≤N, the $\mu_{i}$ is chosen in such a way as to satisfy

(11) $$\sum_{i=1}^{N}{\bar{\rho }}^{i}\left(\lambda^{*}\right)=\sum_{i=1}^{N}\left(\mu_{i}{\bar{\rho }}^{i}\left(\lambda_{-}^{*}\right)+\left(1-\mu_{i}\right){\bar{\rho }}^{i}\left(\lambda_{+}^{*}\right)\right)=M.$$

Then, the mixed policy, denoted by $\phi_{\lambda^{*}}$, is optimal for RP.

**Proof.**  According to Lemma 3.10 of [27], a policy is optimal for RP if

1. It is optimal for $M_{N}\left(\lambda^{*},-1\right)$;
2. The resulting expected transmission rate is equal to *M*.

Then, we construct such a policy using Theorem 2 and Proposition 6. The complete proof can be found in Appendix K.    ☐

Since we approximate $\lambda_{+}^{*}\approx \lambda^{*}+\xi$ and $\lambda_{-}^{*}\approx \lambda^{*}-\xi$ in practice, ${\bar{\rho }}^{i}\left(\lambda_{+}^{*}\right)\leq {\bar{\rho }}^{i}\left(\lambda_{-}^{*}\right)$ for all *i* according to the monotonicity given by Lemma 3.4 of [27]. Combining with the definition of $\lambda^{*}$, we must have $\bar{\rho }\left(\lambda_{+}^{*}\right)\leq M<\bar{\rho }\left(\lambda_{-}^{*}\right)$. Therefore, we can always find $\mu_{i}$’s that realize (11). In this paper, we choose

(12) $$\mu_{i}=\mu =\frac{M-\bar{\rho }\left(\lambda_{+}^{*}\right)}{\bar{\rho }\left(\lambda_{-}^{*}\right)-\bar{\rho }\left(\lambda_{+}^{*}\right)},\;for\;1\leq i\leq N.$$

Then, we describe the algorithm used to obtain the optimal policy for RP. As detailed in Theorem 3, it is essential to find $\lambda^{*}$. To this end, we recall that, for any user *i* under given λ, the optimal deterministic policy $n_{\lambda ,i}$ can be obtained using the results in Section 5.1 and the resulting expected transmission rate ${\bar{\rho }}^{i}\left(\lambda \right)$ is given by Proposition 2. Since ${\bar{\rho }}^{i}\left(\lambda \right)$ is non-increasing in λ for all *i* according to Lemma 3.4 of [27], $\bar{\rho }\left(\lambda \right)=\sum_{i=1}^{N}{\bar{\rho }}^{i}\left(\lambda \right)$ is also non-increasing in λ. Hence, we can regard $\bar{\rho }\left(\lambda \right)$ as a non-increasing function of λ. Then, according to the definition of $\lambda^{*}$, we can use the Bisection search to obtain $\lambda^{*}$ efficiently. The main steps can be summarized as follows.

1. Initialize $\lambda_{-}=0$ and $\lambda_{+}=1$.
2. Do $\lambda_{-}=\lambda_{+}$ and $\lambda_{+}=2\lambda_{+}$ until $\bar{\rho }\left(\lambda_{+}\right)<M$.
3. Run Bisection search on the interval $\left[\lambda_{-},\lambda_{+}\right]$ until the tolerance 2ξ is met.

Then, $\lambda_{-}^{*}$ and $\lambda_{+}^{*}$ can simply be the boundaries of the final interval. The pseudocode for the Bisection search can be found in Algorithm A2 of Appendix M. After obtaining $\lambda_{-}^{*}$ and $\lambda_{+}^{*}$, the optimal policy $\phi_{\lambda^{*}}$ is detailed in Theorem 3 and the mixing probabilities $\mu_{i}$’s are given by (12).

**Remark** **6.** We recall that the optimal deterministic policy for each user can be characterized by two positive thresholds (i.e., $n_{0},n_{1}>0$). Consequently, under RP-optimal policy, the base station will never choose the user at state $\left(0,\hat{r}\right)$. Then, when M increases, the expected transmission rate achieved by RP-optimal policy will saturate before M reaches N. When the expected transmission rate saturates, the RP-optimal policy is $\phi^{*}=\left[n_{1},\ldots ,n_{N}\right]$ where $n_{i}=\left(1,1\right)$ for 1≤i≤N. The saturation happens when M is larger than or equal to the expected transmission rate achieved by $\phi^{*}$.

## 6. Indexed Priority Policy

Although the performance of Whittle’s index policy is known to be good, it requires indexability, which is usually difficult to establish. In this section, based on the primal-dual heuristic introduced in [28], we develop a policy that does not require indexability and has comparable performance to Whittle’s index policy. We start with presenting the primal-dual heuristic.

### 6.1. Primal-Dual Heuristic

The heuristic is based on the optimal primal and dual solution pair to the linear program associated with RP. To introduce the linear program, we define $\pi_{x_{i}}^{a_{i}}\left(\phi \right)\geq 0$ as the expected time that user *i* is at state $x_{i}$ and action $a_{i}$ is taken according to policy ϕ. Then, for any ϕ, $\pi_{x_{i}}^{a_{i}}\left(\phi \right)$ must satisfy the following problems

$$\pi_{x_{i}}^{0}\left(\phi \right)+\pi_{x_{i}}^{1}\left(\phi \right)=\sum_{x_{i}^{\prime }}\sum_{a_{i}^{\prime }}P_{x_{i}^{\prime },x_{i}}\left(a_{i}^{\prime }\right)\pi_{x_{i}^{\prime }}^{a_{i}^{\prime }}\left(\phi \right),\;\forall x_{i},i.$$

$$\sum_{x_{i}}\sum_{a_{i}}\pi_{x_{i}}^{a_{i}}\left(\phi \right)=1,\;\forall i.$$

The objective function of RP can be rewritten as

$$\begin{array}{cc} \underset{\phi \;\in \;\Phi }{\mathrm{minimize}} & \sum_{i=1}^{N}\sum_{x_{i},a_{i}}C(x_{i})\pi_{x_{i}}^{a_{i}}(\phi ), \end{array}$$

where $C\left(x_{i}\right)=f_{i}\left(s_{i}\right)$ is the instant cost at state $x_{i}$. The constraint on the expected transmission rate can be rewritten as

$$\sum_{i=1}^{N}\sum_{x_{i}}\pi_{x_{i}}^{1}\left(\phi \right)\leq M.$$

Thus, the linear program associated with RP can be formulated as the following

(13a)minimizeπxiai∑i=1N∑xi,aiC(xi)πxiai(13b)subject toπxi0 + πxi1 − ∑xi′∑ai′Pxi′,xi(ai′)πxi′ai′ = 0∀xi,i,(13c)∑xi∑aiπxiai = 1∀i,(13d)∑i=1N∑xiπxi1 ≤ M,(13e)πxiai ≥ 0,∀xi,ai,i.

The corresponding dual problem is

(14a)maximizeσ,σi,σxi∑i=1Nσi − Mσ(14b)subject toσxi + σi − ∑xi′Pxi,xi′(0)σxi′ ≤ C(xi),∀xi,i,(14c)σxi + σi − ∑xi′Pxi,xi′(1)σxi′ − σ ≤ C(xi),∀xi,i,(14d)σ ≥ 0.

Let $\left\{{\bar{\pi }}_{x_{i}}^{a_{i}}\right\}$ and $\left\{\bar{\sigma },{\bar{\sigma }}_{i},{\bar{\sigma }}_{x_{i}}\right\}$ be the optimal primal and dual solution pair to the problems reported in (13) and (14). We define

$${\bar{\psi }}_{x_{i}}^{0}=\sum_{x_{i}^{\prime }}P_{x_{i},x_{i}^{\prime }}\left(0\right){\bar{\sigma }}_{x_{i}^{\prime }}+C\left(x_{i}\right)-{\bar{\sigma }}_{i}-{\bar{\sigma }}_{x_{i}}\geq 0,$$

$${\bar{\psi }}_{x_{i}}^{1}=\sum_{x_{i}^{\prime }}P_{x_{i},x_{i}^{\prime }}\left(1\right){\bar{\sigma }}_{x_{i}^{\prime }}+\bar{\sigma }+C\left(x_{i}\right)-{\bar{\sigma }}_{i}-{\bar{\sigma }}_{x_{i}}\geq 0.$$

For any state $x=(x_{1},\ldots ,x_{N})$, let $h\left(x\right)=\sum_{i=1}^{N}{𝟙}_{\left\{{\bar{\pi }}_{x_{i}}^{1}>0\right\}}$. Then, the heuristic operates in the following way

- If h(x)≥M, the base station will choose the *M* users with the largest ${\bar{\psi }}_{x_{i}}^{0}$ among the h(x) users.
- If h(x)<M, these h(x) users are chosen by the base station. The base station will choose M−h(x) additional users with the smallest ${\bar{\psi }}_{x_{i}}^{1}$.

However, Linear Programming (LP) is a very general technique and does not appear to take advantage of the special structure of the problem. Although there are algorithms for solving rational LP that take time polynomial in the number of variables and constraints, they run extremely slowly in practice [29]. For our problem, we notice that the users have separate activity areas that are linked through a common resource constraint. Therefore, the primal problem can be solved using Dantzig-Wolfe decomposition. Even so, the problem is still computationally demanding when the system scales up. We recall that we solved the exact problem efficiently using MDP-specific algorithms in Section 5. It is more efficient because of the following reasons

- According to Proposition 6, we can decompose the problem into *N* subproblems.
- For each subproblem, the threshold structure of the optimal policy is utilized to reduce the running time of RVI.
- As we will see later, the developed policy can be obtained directly from the result of RVI in practice.

In the following, we will translate the results in Section 5 into the optimal primal and dual solution pair and propose Indexed priority policy.

### 6.2. Indexed Priority Policy

We first define the Lagrangian function associated with (13).

$$\begin{array}{cc} L(\pi_{x_{i}}^{a_{i}},\sigma ,\sigma_{i},\sigma_{x_{i}},\psi_{x_{i}}^{a_{i}})= & \left(\sum_{i=1}^{N}\sum_{x_{i},a_{i}}C\left(x_{i}\right)\pi_{x_{i}}^{a_{i}}\right)+\sum_{i,x_{i}}\sigma_{x_{i}}\left(\sum_{x_{i}^{\prime }}\sum_{a_{i}^{\prime }}P_{x_{i}^{\prime },x_{i}}\left(a_{i}^{\prime }\right)\pi_{x_{i}^{\prime }}^{a_{i}^{\prime }}-\pi_{x_{i}}^{0}-\pi_{x_{i}}^{1}\right)+\\ & \sum_{i=1}^{N}\sigma_{i}\left(1-\sum_{x_{i}}\sum_{a_{i}}\pi_{x_{i}}^{a_{i}}\right)+\sigma \left(\sum_{i=1}^{N}\sum_{x_{i}}\pi_{x_{i}}^{1}-M\right)-\sum_{i,x_{i},a_{i}}\psi_{x_{i}}^{a_{i}}\pi_{x_{i}}^{a_{i}}. \end{array}$$

Then, the corresponding Lagrangian dual function is

$$g\left(\sigma ,\sigma_{i},\sigma_{x_{i}},\psi_{x_{i}}^{a_{i}}\right)=\underset{\pi_{x_{i}}^{a_{i}}}{\inf}L\left(\pi_{x_{i}}^{a_{i}},\sigma ,\sigma_{i},\sigma_{x_{i}},\psi_{x_{i}}^{a_{i}}\right).$$

Let $\pi_{x_{i}}$ be the expected time that user *i* is at state $x_{i}$ caused by the adoption of $\phi_{\lambda^{*}}$, where $\phi_{\lambda^{*}}$ is the optimal policy detailed in Theorem 3. Then, we define $\left\{\pi_{x_{i}}^{a_{i}}\right\}$ as follows

- State $x_{i}$ is where randomization happens (randomization happens when the actions suggested by the two optimal deterministic policies are different), and it has a value of $\pi_{x_{i}}^{0}=a_{n_{\lambda_{-}^{*},i}}\left(x_{i}\right)\left(1-\mu_{i}\right)\pi_{x_{i}}+a_{n_{\lambda_{+}^{*},i}}\left(x_{i}\right)\mu_{i}\pi_{x_{i}}$ and $\pi_{x_{i}}^{1}=\pi_{x_{i}}-\pi_{x_{i}}^{0}$ where $\mu_{i}$ is given by (12) and $a_{n_{\lambda ,i}}\left(x_{i}\right)$ is the action suggested by $n_{\lambda ,i}$ at state $x_{i}$.
- For other values of $x_{i}$, we have $\pi_{x_{i}}^{0}=\left(1-a_{n_{\lambda^{*},i}}\left(x_{i}\right)\right)\pi_{x_{i}}$ and $\pi_{x_{i}}^{1}=\pi_{x_{i}}-\pi_{x_{i}}^{0}$.

We also define $\sigma =\lambda^{*}$, $\sigma_{i}=\theta_{i}$, and $\sigma_{x_{i}}=V^{i}\left(x_{i}\right)$ where $\lambda^{*}$ is specified in Section 5.2, $\theta_{i}$ is the optimal value of $M_{1}^{i}\left(\lambda^{*},-1\right)$, and $V^{i}\left(x_{i}\right)$ is the value function associated with $M_{1}^{i}\left(\lambda^{*},-1\right)$. Lastly, we define $\left\{\psi_{x_{i}}^{a_{i}}\right\}$ as follows

$$\psi_{x_{i}}^{0}=\sum_{x_{i}^{\prime }}P_{x_{i},x_{i}^{\prime }}\left(0\right)\sigma_{x_{i}^{\prime }}+C\left(x_{i}\right)-\sigma_{i}-\sigma_{x_{i}},$$

$$\psi_{x_{i}}^{1}=\sum_{x_{i}^{\prime }}P_{x_{i},x_{i}^{\prime }}\left(1\right)\sigma_{x_{i}^{\prime }}+\sigma +C\left(x_{i}\right)-\sigma_{i}-\sigma_{x_{i}}.$$

Then, we can prove the following proposition.

**Proposition** **7** (Optimal solution pair)**.** *$\left\{\pi_{x_{i}}^{a_{i}}\right\}$ and $\left\{\sigma ,\sigma_{i},\sigma_{x_{i}},\psi_{x_{i}}^{a_{i}}\right\}$ are primal and dual solutions to *(13)*, respectively.*

**Proof.**  Since (13) is linear and strictly feasible, it is sufficient to show that $\left\{\pi_{x_{i}}^{a_{i}}\right\}$ and $\left\{\sigma ,\sigma_{i},\sigma_{x_{i}},\psi_{x_{i}}^{a_{i}}\right\}$ verify the KKT conditions, which can be expressed as the following four conditions.

1. Primal feasibility: the constraints in (13) are satisfied.
2. Dual feasibility: σ≥0 and $\psi_{x_{i}}^{a_{i}}\geq 0$ for all $x_{i}$, $a_{i}$, and *i*.
3. Complementary slackness: $\sigma \left(\sum_{i=1}^{N}\sum_{x_{i}}\pi_{x_{i}}^{1}-M\right)=0$ and $\psi_{x_{i}}^{a_{i}}\pi_{x_{i}}^{a_{i}}=0$ for all $x_{i}$, $a_{i}$, and *i*.
4. Stationarity: the gradient of $L(\pi_{x_{i}}^{a_{i}},\sigma ,\sigma_{i},\sigma_{x_{i}},\psi_{x_{i}}^{a_{i}})$ with respect to $\left\{\pi_{x_{i}}^{a_{i}}\right\}$ vanishes.

Apparently, the first condition is satisfied by $\left\{\pi_{x_{i}}^{a_{i}}\right\}$. For the second condition, σ≥0 since $\sigma =\lambda^{*}\geq 0$ by definition. For $\psi_{x_{i}}^{a_{i}}$, we can verify that $\psi_{x_{i}}^{a_{i}}=V^{i,a_{i}}\left(x_{i}\right)-V^{i}\left(x_{i}\right)$ where $V^{i,a_{i}}\left(x_{i}\right)$ is the value function resulting from taking action $a_{i}$ at state $x_{i}$. Then, the non-negativity is guaranteed by the Bellman equation. For the third condition, the first term is zero because we choose the $\mu_{i}$’s given by (12). For the second term, we recall that $\psi_{x_{i}}^{a_{i}}=V^{i,a_{i}}\left(x_{i}\right)-V^{i}\left(x_{i}\right)$. According to the definition of $\pi_{x_{i}}^{a_{i}}$, we know $V^{i}\left(x_{i}\right)=V^{i,a_{i}}\left(x_{i}\right)$ if $\pi_{x_{i}}^{a_{i}}>0$. Combined together, we can conclude that $\psi_{x_{i}}^{a_{i}}=0$ when $\pi_{x_{i}}^{a_{i}}>0$. Thus, the third condition is satisfied. For the last condition, setting the gradient equal to zero yields a system of linear equations. More precisely, for each $x_{i}$ and 1≤i≤N

$$\left\{ \begin{array}{c} \sum_{x_{i}^{\prime }}P_{x_{i},x_{i}^{\prime }}\left(0\right)\sigma_{x_{i}^{\prime }}+C\left(x_{i}\right)=\sigma_{x_{i}}+\sigma_{i}+\psi_{x_{i}}^{0}.\\ \sum_{x_{i}^{\prime }}P_{x_{i},x_{i}^{\prime }}\left(1\right)\sigma_{x_{i}^{\prime }}+\sigma +C\left(x_{i}\right)=\sigma_{x_{i}}+\sigma_{i}+\psi_{x_{i}}^{1}. \end{array}\right.$$

Then, $\left\{\sigma ,\sigma_{i},\sigma_{x_{i}},\psi_{x_{i}}^{a_{i}}\right\}$ verifies the system of linear equations by definition. Since all four conditions are satisfied, we can conclude our proof.    ☐

According to Proposition 7, we know that $\left\{\pi_{x_{i}}^{a_{i}}\right\}$ and $\left\{\sigma ,\sigma_{i},\sigma_{x_{i}}\right\}$ defined above are the optimal solutions to problems (13) and (14), respectively. As the optimal solutions are obtained, we can adopt the heuristic detailed in Section 6.1.

The heuristic can be expressed equivalently as an index policy. To this end, we define the index $I_{x_{i}}$ for state $x_{i}$ as

$$I_{x_{i}}≜{\bar{\psi }}_{x_{i}}^{0}-{\bar{\psi }}_{x_{i}}^{1}.$$

According to the complementary slackness, $I_{x_{i}}$ can be reduced to the following.

- For state $x_{i}$ such that ${\bar{\pi }}_{x_{i}}^{1}>0$ and ${\bar{\pi }}_{x_{i}}^{0}=0$, we have ${\bar{\psi }}_{x_{i}}^{1}=0$. Therefore, $I_{x_{i}}={\bar{\psi }}_{x_{i}}^{0}\geq 0$.
- For state $x_{i}$ such that ${\bar{\pi }}_{x_{i}}^{1}>0$ and ${\bar{\pi }}_{x_{i}}^{0}>0$, we have ${\bar{\psi }}_{x_{i}}^{1}={\bar{\psi }}_{x_{i}}^{0}=0$. Therefore, $I_{x_{i}}=0$.
- For state $x_{i}$ such that ${\bar{\pi }}_{x_{i}}^{1}=0$ and ${\bar{\pi }}_{x_{i}}^{0}>0$, we have ${\bar{\psi }}_{x_{i}}^{0}=0$. Therefore, $I_{x_{i}}=-{\bar{\psi }}_{x_{i}}^{1}\leq 0$.

We can show that $I_{x_{i}}$ possesses the following properties.

**Proposition** **8** (Properties of $I_{x_{i}}$)**.** For 1≤i≤N, $I_{x_{i}}\geq -\lambda^{*}$ for any $x_{i}$. The equality holds when ${\hat{r}}_{i}=p_{e,i}^{0}=0$ or $s_{i}=0$. At the same time, $I_{x_{i}}$ is non-decreasing in both $s_{i}$ and ${\hat{r}}_{i}$.

**Proof.**  We notice that $I_{x_{i}}$ can be expressed as a function of $V^{i}\left(x_{i}\right)$ and $\lambda^{*}$. Meanwhile, $M_{1}^{i}\left(\lambda^{*},-1\right)$ coincides with the decoupled model studied in Section 4.2. Then, we can verify the properties of $I_{x_{i}}$ using the results in Section 4.2. The complete proof can be found in Appendix L.    ☐

Comparing with the heuristic detailed in Section 6.1, we can define the Indexed priority policy.

**Definition** **5** (Indexed priority policy)**.** *At any state $x=(x_{1},x_{2},\ldots ,x_{N})$, the base station will transmit the updates from M users with the largest $I_{x_{i}}$. The ties are broken arbitrarily.*

**Remark** **7.** *Indexed priority policy belongs to the class of priority policies introduced in [30]. These priority policies are asymptotically optimal when certain conditions are satisfied.*

**Remark** **8.** *Indexed priority policy possesses the structural properties detailed in Corollary 1.*

- The first two properties can be verified by noting that $I_{x_{i}}\geq -\lambda^{*}$ and the equality holds when ${\hat{r}}_{i}=p_{e,i}^{0}=0$ or $s_{i}=0$. At the same time, $I_{x_{i}}$ is non-decreasing in ${\hat{r}}_{i}$.
- *The third and fourth properties can be verified by noting that $I_{x_{i}}$ is non-decreasing in $s_{i}$.*
- *For the last property, we first notice that $I_{x_{j}}=I_{x_{k}}$ when users j and k are statistically identical and $x_{j}=x_{k}$. Then, the property can be verified by noting that $I_{x_{i}}$ is non-decreasing in both $s_{i}$ and ${\hat{r}}_{i}$.*

We notice that $\theta_{i}$’s and $C(x_{i})$’s are canceled out by the definition of $I_{x_{i}}$. Therefore, $I_{x_{i}}$ can be calculated using $\lambda^{*}$ and the value function of $M_{1}^{i}\left(\lambda^{*},-1\right)$. In practice, we can use either $\lambda_{-}^{*}$ or $\lambda_{+}^{*}$ to approximate $\lambda^{*}$, and the value function can be approximated by the result of the RVI detailed in Section 5.1. Since the state space is infinite, we only calculate a finite number of $V^{i}\left(x_{i}\right)$, the number of which depends on the truncation parameter *m* of ASM. Meanwhile, the probabilities $P_{x_{i},x_{i}^{\prime }}\left(a_{i}\right)$ in $I_{x_{i}}$ are modified according to (10).

## 7. Numerical Results

In this section, we provide numerical results to showcase the performance of the developed scheduling policies. To eliminate the effect of *N*, we plot the expected average AoII. In particular, we provide the expected average AoII achieved by the Indexed priority policy and Whittle’s index policy when M=1. The policies are calculated using the results detailed in Section 4, Section 5 and Section 6. When obtaining the Indexed priority policy, we set the tolerance in the Bisection search to ξ=0.005. Meanwhile, we choose the truncation parameter in ASM m=800 and the convergence criteria in RVI ϵ=0.01. We notice that the calculation of Whittle’s index involves an infinite sum. In practice, we approximate the result by replacing +∞ with a large enough number $k_{max}$. Here, we choose $k_{max}=800$. For both scheduling policies, the resulting expected average AoII is obtained via simulations. Each data point is the average of 15 runs with 15,000 time slots considered in each run.

We also compare the developed policies with the optimal policy for RP, which can be calculated by following the discussion in Section 5.2. We adopt the same choices of parameters as we used to obtain the developed policies. The corresponding performance is calculated using Proposition 2. Like before, the infinite sum is approximated by replacing +∞ with $k_{max}=800$. We also provide the expected average AoII achieved by the Greedy policy to show the performance advantages of the developed policies. When the Greedy policy is adopted, the base station always chooses the user with the largest AoII. The resulting expected average AoII is obtained via the same simulations as applied to the developed policies.

**Figure 3:**
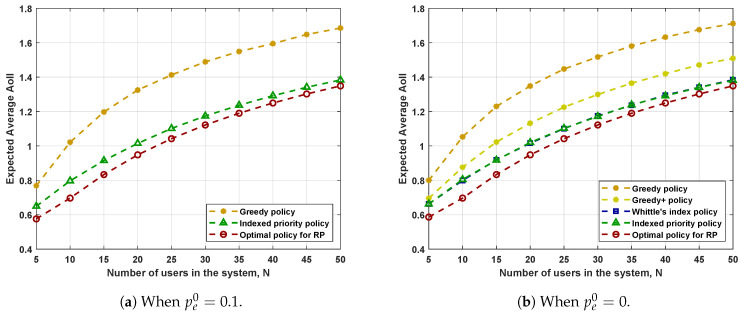
Performance when the source processes vary. We choose $p_{i}=0.05+\frac{0.4(i-1)}{N-1}$, $f_{i}\left(s\right)=s$, $\gamma_{i}=0.6$, $p_{e,i}^{0}=p_{e}^{0}$, and $p_{e,i}^{1}=0.1$ for 1≤i≤N.

**Figure 4:**
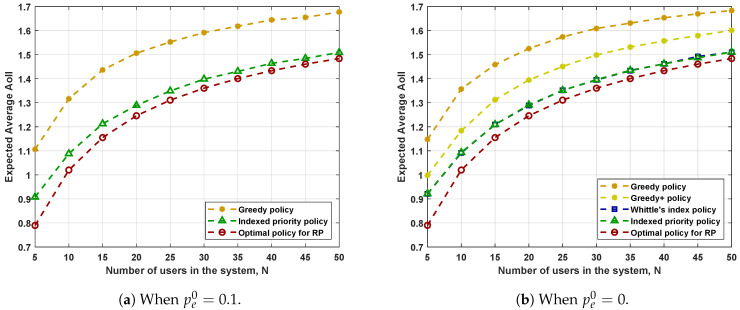
Performance when the communication goals vary. We choose $f_{i}\left(s\right)=s^{0.5+\frac{i-1}{N-1}}$, $p_{i}=0.3$, $\gamma_{i}=0.6$, $p_{e,i}^{0}=p_{e}^{0}$, and $p_{e,i}^{1}=0.1$ for 1≤i≤N.

Figure 3 and Figure 4 illustrate the performance when the source processes have different dynamics and when each user’s communication goal is different, respectively. Figure 3a provides the performance when $p_{i}=0.05+\frac{0.4(i-1)}{N-1}$ for 1≤i≤N. For other parameters, the users make the same choices. More precisely, $f_{i}\left(s\right)=s$, $\gamma_{i}=0.6$, and $p_{e,i}^{0}=p_{e,i}^{1}=0.1$ for 1≤i≤N. Figure 4a provides the performance when $f_{i}\left(s\right)=s^{0.5+\frac{i-1}{N-1}}$ for 1≤i≤N. Same as before, the users make the same choices for other parameters. More precisely, $p_{i}=0.3$, $\gamma_{i}=0.6$, and $p_{e,i}^{0}=p_{e,i}^{1}=0.1$ for 1≤i≤N. In Figure 3b and Figure 4b, we force $p_{e,i}^{0}=0$ for all users to ensure the existence of Whittle’s index. Other choices remain the same as in Figure 3a and Figure 4a. According to Corollary 1, the optimal policy will never choose the user with $\hat{r}=p_{e}^{0}=0$ unless it is to break the tie. Therefore, in Figure 3b and Figure 4b, we also consider the Greedy+ policy where the base station always chooses the user with the largest AoII among the users with $\hat{r}=1$. The resulting expected average AoII is obtained via the same simulations as applied to the Greedy policy.

**Figure 5:**
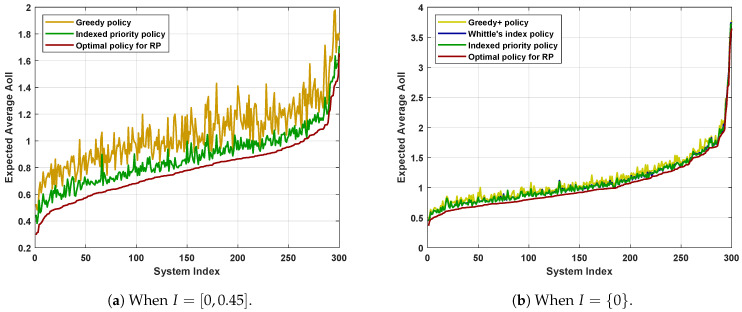
Performance in systems with random parameters when N=5. The parameters for each user are chosen randomly within the following intervals: γ∈[0,1], p∈[0.05,0.45], $p_{e}^{0}\in I$, $p_{e}^{1}\in \left[0,0.45\right]$, and $f\left(s\right)=s^{\tau }$ where τ∈[0.5,1.5].

Figure 5 shows the performance in systems where the parameters for each user are generated uniformly and randomly within their ranges. In Figure 5a, we consider N=5, γ∈[0,1], p∈[0.05,0.45], $p_{e}^{\hat{r}}\in \left[0,0.45\right]$, and $f\left(s\right)=s^{\tau }$, where τ∈[0.5,1.5]. There are a total of 300 different choices and the results are sorted by the performance of RP-optimal policy in ascending order. Figure 5b adopts the same system settings except that we impose $p_{e,i}^{0}=0$ for 1≤i≤N to ensure the feasibility of Whittle’s index policy. Meanwhile, we ignore the Greedy policy since the Greedy+ policy achieves a better performance, as indicated by Figure 3b and Figure 4b.

We can make the following observations from the figures.

- The Greedy+ policy yields a smaller expected average AoII than that achieved by the Greedy policy. Recall that we obtained the Greedy+ policy by applying the structural properties detailed in Corollary 1. Therefore, simple applications of the structural properties of the optimal policy can improve the performance of scheduling policies.
- The Indexed priority policy has comparable performance to Whittle’s index policy in all the system settings considered. The two policies have their own advantages. The Indexed priority policy has a broader scope of application, while Whittle’s index policy has a lower computational complexity.
- The performance of the Indexed priority policy and Whittle’s index policy is better than that of the Greedy/Greedy+ policies and is not far from the performance of the RP-optimal policy. Recall that the performance of the RP-optimal policy forms a universal lower bound on the performance of all admissible policies for PP. Hence, we can conclude that both the Indexed priority policy and Whittle’s index policy achieve good performances.

## 8. Conclusions

In this paper, we studied the problem of minimizing the Age of Incorrect Information in a slotted-time system where a base station needs to schedule *M* users among *N* available users. Meanwhile, the base station has access to imperfect channel state information in each time slot. The problem is a restless multi-armed bandit problem which is SPACE-hard. However, by casting the problem into a Markov decision process, we obtain the structural properties of the optimal policy. Then, we introduce a relaxed version of the original problem and investigate the decoupled model. Under a simple condition, we establish the indexability of the decoupled problem and obtain the expression of Whittle’s index. On this basis, we developed Whittle’s index policy. To get rid of the requirement for indexability, we developed the Indexed priority policy based on the optimal policy for the relaxed problem. The characteristics of the relaxed problem are explored to make the calculation of its optimal policy more efficient. Finally, through numerical results, we show that simple applications of the structural properties can improve the performance of scheduling policies. Moreover, Whittle’s index policy and the Indexed priority policy achieve good and comparable performances.

## Figures and Tables

**Figure A1:**
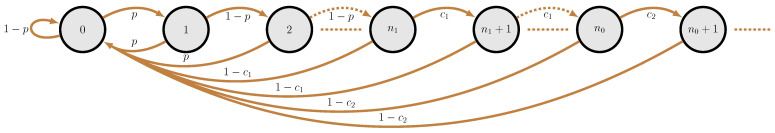
DTMC induced by the threshold policy $n=(n_{0},n_{1})$. In the figure, $c_{1}=\left(1-\gamma \right)\left(1-p\right)+\gamma \alpha$ and $c_{2}=\left(1-\gamma \right)$β+γα.

## Data Availability

Not applicable.

## Appendix A. Proof of Lemma 1

We consider two states, $x_{1}$ and $x_{2}$, that differ only in the value of $s_{j}$. Without the loss of generality, we assume $s_{1,j}<s_{2,j}$. Then, it is sufficient to show that, for any 1≤j≤N, $V\left(x_{1}\right)\leq V\left(x_{2}\right)$. Leveraging the iterative nature of VIA, we use mathematical induction to prove the monotonicity. First of all, the base case (i.e., ν=0) is true by initialization. We assume the lemma holds at iteration ν. Then, we want to examine whether it holds at iteration ν+1. The update step reported in problem (5) can be rewritten as follows.

(A1) $$V_{\nu +1}\left(x\right)=\min_{a\in A_{N}\left(1\right)}V_{\nu +1}^{a}\left(x\right),$$

where

$$\begin{array}{cc} V_{\nu +1}^{a}\left(x\right) & =C\left(x\right)-\theta +\sum_{x^{\prime }-\left\{x_{j}^{\prime }\right\}}\left\{\left(\prod_{i\neq j}P_{x_{i},x_{i}^{\prime }}\left(a_{i}\right)\right)\sum_{{\hat{r}}_{j}^{\prime }}P\left({\hat{r}}_{j}^{\prime }\right)U_{\nu }^{j}\left(x,x^{\prime }\right)\right\}, \end{array}$$

$$U_{\nu }^{j}\left(x,x^{\prime }\right)=\sum_{s_{j}^{\prime }}P_{s_{j},s_{j}^{\prime }}\left(a_{j},{\hat{r}}_{j}\right)V_{\nu }\left(x^{\prime }\right).$$

To prove the desired results, we distinguish between the following cases.

- We first consider the case of $s_{1,j}=0<s_{2,j}$ and ${\hat{r}}_{1,j}={\hat{r}}_{2,j}=0$. When $a_{j}=1$ and for any $x^{\prime }-\left\{s_{j}^{\prime }\right\}$, we have
  $$U_{\nu }^{j}\left(x_{1},x^{\prime }\right)=p_{j}V_{\nu }\left(x^{\prime };s_{j}^{\prime }=1\right)+\left(1-p_{j}\right)V_{\nu }\left(x^{\prime };s_{j}^{\prime }=0\right),$$
  $$\begin{array}{cc} U_{\nu }^{j}\left(x_{2},x^{\prime }\right) & =\beta_{j}V_{\nu }\left(x^{\prime };s_{j}^{\prime }=s_{2,j}+1\right)+\left(1-\beta_{j}\right)V_{\nu }\left(x^{\prime };s_{j}^{\prime }=0\right), \end{array}$$
  where $V_{\nu }\left(x^{\prime };s_{j}^{\prime }=0\right)$ is the estimated value function of the state $x^{\prime }$ with $s_{j}^{\prime }=0$ at iteration ν (at the risk of abusing the notation, we use $V(x;s_{j}=s_{1})$ and $V(x;s_{j}=s_{2})$ to represent the value functions of two states that differ only in the value of $s_{j}$). Then, we get
  $$\begin{array}{c} U_{\nu }^{j}\left(x_{1},x^{\prime }\right)-U_{\nu }^{j}\left(x_{2},x^{\prime }\right)\leq \left(p_{j}-\beta_{j}\right)(V_{\nu }\left(x^{\prime };s_{j}^{\prime }=1\right)-V_{\nu }\left(x^{\prime };s_{j}^{\prime }=0\right))\leq 0. \end{array}$$
  The inequalities hold since $\beta_{j}>p_{j}$ and Lemma 1 are true at iteration ν by assumption. Therefore, we have $U_{\nu }^{j}\left(x_{1},x^{\prime }\right)\leq U_{\nu }^{j}\left(x_{2},x^{\prime }\right)$ when $a_{j}=1$ for any $x^{\prime }-\left\{s_{j}^{\prime }\right\}$.
  For the case of $a_{i}=1$ where i≠j, we notice that $a_{j}=0$. Then, for any $x^{\prime }-\left\{s_{j}^{\prime }\right\}$, we obtain
  $$U_{\nu }^{j}\left(x_{1},x^{\prime }\right)=p_{j}V_{\nu }\left(x^{\prime };s_{j}^{\prime }=1\right)+\left(1-p_{j}\right)V_{\nu }\left(x^{\prime };s_{j}^{\prime }=0\right),$$
  $$\begin{array}{cc} U_{\nu }^{j}\left(x_{2},x^{\prime }\right) & =\left(1-p_{j}\right)V_{\nu }\left(x^{\prime };s_{j}^{\prime }=s_{2,j}+1\right)+p_{j}V_{\nu }\left(x^{\prime };s_{j}^{\prime }=0\right). \end{array}$$
  Therefore, when $a_{i}=1$, we have
  $$\begin{array}{c} U_{\nu }^{j}\left(x_{1},x^{\prime }\right)-U_{\nu }^{j}\left(x_{2},x^{\prime }\right)\leq \left(2p_{j}-1\right)(V_{\nu }\left(x^{\prime };s_{j}^{\prime }=1\right)-V_{\nu }\left(x^{\prime };s_{j}^{\prime }=0\right))\leq 0. \end{array}$$
  The inequalities hold since $2p_{j}-1<0$ and Lemma 1 is true at iteration ν by assumption. Combining with the case of $a_{j}=1$, $U_{\nu }^{j}\left(x_{1},x^{\prime }\right)\leq U_{\nu }^{j}\left(x_{2},x^{\prime }\right)$ holds for any $x^{\prime }-\left\{s_{j}^{\prime }\right\}$ under any feasible action. Since $x_{1}$ and $x_{2}$ differ only in the value of $s_{j}$ and C(x) is non-decreasing in $s_{i}$ for 1≤i≤N, we can see that $V_{\nu +1}^{a}\left(x_{1}\right)\leq V_{\nu +1}^{a}\left(x_{2}\right)$ for any feasible a. Then, by (A1), we can conclude that the lemma holds at iteration ν+1 when $s_{1,j}=0<s_{2,j}$ and ${\hat{r}}_{1,j}={\hat{r}}_{2,j}=0$.
- When $s_{1,j}=0<s_{2,j}$ and ${\hat{r}}_{1,j}={\hat{r}}_{2,j}=1$, by replacing the $\beta_{j}$’s in the above case with $\alpha_{j}$’s, we can achieve the same result.
- When $0<s_{1,j}<s_{2,j}$ and ${\hat{r}}_{1,j}={\hat{r}}_{2,j}$, we notice that
  $$\begin{array}{cc} & P_{s_{1,j},s_{1,j}+1}\left(a_{j},{\hat{r}}_{1,j}\right)=P_{s_{2,j},s_{2,j}+1}\left(a_{j},{\hat{r}}_{2,j}\right),\\ & P_{s_{1,j},0}\left(a_{j},{\hat{r}}_{1,j}\right)=P_{s_{2,j},0}\left(a_{j},{\hat{r}}_{2,j}\right). \end{array}$$
  Then, leveraging the monotonicity of $V_{\nu }\left(x\right)$ and C(x), we can conclude with the same result.

Combining the three cases, we prove that the lemma also holds at iteration ν+1 of VIA. Therefore, the lemma holds at any iteration ν by mathematical induction. Since the results hold for any 1≤j≤N and VIA is guaranteed to converge to the value function when ν→+∞, we can conclude our proof.

## Appendix B. Proof of Lemma 2

We inherit the notations in the proof of Lemma 1. We still use mathematical induction to obtain the desired results. The base case ν=0 is true by initialization. We assume the lemma holds at iterative ν and examine whether it still holds at iteration ν+1. In the case of M=1, we rewrite (5) as

(A2) $$V_{\nu +1}\left(x\right)=\min_{1\leq j\leq N}V_{\nu +1}^{j}\left(x\right),$$

where

(A3) $$V_{\nu +1}^{j}\left(x\right)=C\left(x\right)-\theta +\sum_{x^{\prime }}\left\{\left(\prod_{i\neq j}P_{x_{i},x_{i}^{\prime }}^{i}\left(0\right)\right)P_{x_{j},x_{j}^{\prime }}^{j}\left(1\right)V_{\nu }\left(x^{\prime }\right)\right\},$$

and $P_{x,x^{\prime }}^{i}\left(a_{i}\right)$ is the probability that action $a_{i}$ will lead to state $x^{\prime }$ when user *i* is at state *x*. To get the desired results, we distinguish between the following cases

- We first show that $V_{\nu +1}^{j}\left(x\right)=V_{\nu +1}^{k}\left(P\left(x\right)\right)$. According to (A3), we have
  $$V_{\nu +1}^{j}\left(x\right)=C\left(x\right)-\theta +\sum_{x^{\prime }}\left\{\left(\prod_{i\neq j,k}P_{x_{i},x_{i}^{\prime }}^{i}\left(0\right)\right)P_{x_{k},x_{k}^{\prime }}^{k}\left(0\right)P_{x_{j},x_{j}^{\prime }}^{j}\left(1\right)V_{\nu }\left(x^{\prime }\right)\right\}.$$
  $$\begin{array}{cc} V_{\nu +1}^{k}\left(P\left(x\right)\right) & =C(P(x))-\theta +\\ & \sum_{P{\left(x\right)}^{\prime }}\left(\prod_{i\neq j,k}P_{P{\left(x\right)}_{i},P{\left(x\right)}_{i}^{\prime }}^{i}\left(0\right)\right)P_{P{\left(x\right)}_{k},P{\left(x\right)}_{k}^{\prime }}^{k}\left(1\right)P_{P{\left(x\right)}_{j},P{\left(x\right)}_{j}^{\prime }}^{j}\left(0\right)V_{\nu }\left(P{\left(x\right)}^{\prime }\right). \end{array}$$
  It is obvious that for any $P{\left(x\right)}^{\prime }$, there always exists $P\left(x^{\prime \prime }\right)=P{\left(x\right)}^{\prime }$. Then, we obtain
  $$\begin{array}{cc} V_{\nu +1}^{k}\left(P\left(x\right)\right) & =C(P(x))-\theta +\\ & \;\;\;\sum_{P(x^{\prime \prime })}\left(\prod_{i\neq j,k}P_{x_{i},x_{i}^{\prime \prime }}^{i}\left(0\right)\right)P_{x_{j},P{\left(x^{\prime \prime }\right)}_{k}}^{k}\left(1\right)P_{x_{k},P{\left(x^{\prime \prime }\right)}_{j}}^{j}\left(0\right)V_{\nu }\left(P\left(x^{\prime \prime }\right)\right)\\ & =C\left(P\left(x\right)\right)-\theta +\sum_{x^{\prime \prime }}\left(\prod_{i\neq j,k}P_{x_{i},x_{i}^{\prime \prime }}^{i}\left(0\right)\right)P_{x_{j},x_{j}^{\prime \prime }}^{k}\left(1\right)P_{x_{k},x_{k}^{\prime \prime }}^{j}\left(0\right)V_{\nu }\left(x^{\prime \prime }\right)\\ & =C\left(P\left(x\right)\right)-\theta +\sum_{x^{\prime }}\left(\prod_{i\neq j,k}P_{x_{i},x_{i}^{\prime }}^{i}\left(0\right)\right)P_{x_{j},x_{j}^{\prime }}^{k}\left(1\right)P_{x_{k},x_{k}^{\prime }}^{j}\left(0\right)V_{\nu }\left(x^{\prime }\right). \end{array}$$
  The second equality follows from the definition of P(·), the property of summation, and the assumption at iteration ν. The last equality follows from the variable renaming. Then, by the definition of statistically identical, we have $P_{x_{j},x_{j}^{\prime }}^{k}\left(1\right)=P_{x_{j},x_{j}^{\prime }}^{j}\left(1\right)$, $P_{x_{k},x_{k}^{\prime }}^{j}\left(0\right)=P_{x_{k},x_{k}^{\prime }}^{k}\left(0\right)$, and C(x)=C(P(x)). Therefore, we can conclude that $V_{\nu +1}^{j}\left(x\right)=V_{\nu +1}^{k}\left(P\left(x\right)\right)$.
- Along the same lines, we can easily show that $V_{\nu +1}^{k}\left(x\right)=V_{\nu +1}^{j}\left(P\left(x\right)\right)$ and $V_{\nu +1}^{i}\left(x\right)=V_{\nu +1}^{i}\left(P\left(x\right)\right)$ for i≠j,k.

Combining the above cases with (A2), we prove that $V_{\nu +1}\left(x\right)=V_{\nu +1}\left(P\left(x\right)\right)$. Then, by induction, we have $V_{\nu }\left(x\right)=V_{\nu }\left(P\left(x\right)\right)$ at any iteration ν. Since VIA is guaranteed to converge to the value function when ν→+∞, we can conclude our proof.

## Appendix C. Proof of Theorem 1

For arbitrary *j* and *k*

(A4) $$\delta^{j,k}\left(x\right)=\sum_{x^{\prime }-\left\{x_{j}^{\prime },x_{k}^{\prime }\right\}}\left\{\left(\prod_{i\neq j,k}P_{x_{i},x_{i}^{\prime }}\left(0\right)\right)\sum_{{\hat{r}}_{j}^{\prime },{\hat{r}}_{k}^{\prime }}P\left({\hat{r}}_{j}^{\prime }\right)P\left({\hat{r}}_{k}^{\prime }\right)R^{j,k}\left(x,x^{\prime }\right)\right\},$$

where

(A5) $$R^{j,k}\left(x,x^{\prime }\right)=\sum_{s_{j}^{\prime },s_{k}^{\prime }}\left[\left(P_{s_{k},s_{k}^{\prime }}\left(0,{\hat{r}}_{k}\right)P_{s_{j},s_{j}^{\prime }}\left(1,{\hat{r}}_{j}\right)-P_{s_{k},s_{k}^{\prime }}\left(1,{\hat{r}}_{k}\right)P_{s_{j},s_{j}^{\prime }}\left(0,{\hat{r}}_{j}\right)\right)V\left(x^{\prime }\right)\right].$$

With this in mind, we will prove the properties one by one.

Property 1—*δ*^*j*,*k*^ (***x***) ≤ 0 if ${\hat{r}}_{k}\;=\;p_{e,k}^{0}\;=\;0$. The equality holds when $s_{j}\;=\;0$ or ${\hat{r}}_{j}\;=\;p_{e,j}^{0}\;=\;0$.

When ${\hat{r}}_{k}=p_{e,k}^{0}=0$, transmitting the update from user *k* will necessarily fail. Therefore, $P_{s_{k},s_{k}^{\prime }}\left(0,0\right)=P_{s_{k},s_{k}^{\prime }}\left(1,0\right)$ for any $s_{k}$ and $s_{k}^{\prime }$. Then, we have

$$\begin{array}{c} R^{j,k}\left(x,x^{\prime }\right)=\sum_{s_{k}^{\prime }}P_{s_{k},s_{k}^{\prime }}\left(0,0\right)\sum_{s_{j}^{\prime }}\left[\left(P_{s_{j},s_{j}^{\prime }}\left(1,{\hat{r}}_{j}\right)-P_{s_{j},s_{j}^{\prime }}\left(0,{\hat{r}}_{j}\right)\right)V\left(x^{\prime }\right)\right]. \end{array}$$

To identify the sign of $R^{j,k}\left(x,x^{\prime }\right)$, we distinguish between the following cases

- When $s_{j}=0$, we can easily show that $R^{j,k}\left(x,x^{\prime }\right)=0$ for any $x^{\prime }-\left\{s_{j}^{\prime },s_{k}^{\prime }\right\}$ by noticing that the two possible actions with respect to user *j* (i.e., $a_{j}=1$ and $a_{j}=0$) are equivalent when $s_{j}=0$. Since $\delta^{j,k}\left(x\right)$ is a linear combination of $R^{j,k}\left(x,x^{\prime }\right)$’s with non-negative coefficients, we can conclude that $\delta^{j,k}\left(x\right)=0$ in this case.
- When $s_{j}>0$ and ${\hat{r}}_{j}=1$, for any $x^{\prime }-\left\{s_{j}^{\prime },s_{k}^{\prime }\right\}$, we have
  (A6) $$\begin{array}{cc} R^{j,k}\left(x,x^{\prime }\right) & =\sum_{s_{k}^{\prime }}P_{s_{k},s_{k}^{\prime }}\left(0,0\right)\left(\alpha_{j}+p_{j}-1\right)\left(V\left(x^{\prime };s_{j}^{\prime }=s_{j}+1\right)-V\left(x^{\prime };s_{j}^{\prime }=0\right)\right)\\ \; & \leq 0. \end{array}$$
  The inequality holds because of Lemma 1 and the fact that $\alpha_{j}+p_{j}<1$. We recall that $\delta^{j,k}\left(x\right)$ is a linear combination of $R^{j,k}\left(x,x^{\prime }\right)$’s with non-negative coefficients. Then, we can conclude that $\delta^{j,k}\left(x\right)\leq 0$ in this case.
- When $s_{j}>0$ and ${\hat{r}}_{j}=0$, by replacing the $\alpha_{j}$ in (A6) with $\beta_{j}$, we can get the same result. In this case, the equality holds when $\beta_{j}+p_{j}=1$, or, equivalently, $p_{e,j}^{0}=0$.

Combining the cases, we prove the first property.

Property 2—*δ*^*j*,*k*^ (***x***) is non-increasing in ${\hat{r}}_{j}$ and is non-decreasing in ${\hat{r}}_{k}$ when $s_{j},s_{k}\;>\;0$. At the same time, $\delta^{j,k}\left(x\right)$ is independent of ${\hat{r}}_{j}$ for any *i* ≠ *j*,*k*.

We first prove the monotonicity of $\delta^{j,k}\left(x\right)$ with respect to ${\hat{r}}_{j}$. To this end, we define $x_{1}$ and $x_{2}$ as two states that differ only in the value of ${\hat{r}}_{j}$. Without a loss of generality, we assume ${\hat{r}}_{1,j}=1$ and ${\hat{r}}_{2,j}=0$. Then, we investigate the sign of $\delta^{j,k}\left(x_{1}\right)-\delta^{j,k}\left(x_{2}\right)$. We define $x_{i}≜x_{1,i}=x_{2,i}$ for i≠j. Then, according to (A4), $\delta^{j,k}\left(x_{1}\right)-\delta^{j,k}\left(x_{2}\right)$ can be written as

$$\begin{array}{cc} \delta^{j,k}\left(x_{1}\right)- & \delta^{j,k}\left(x_{2}\right)=\\ & \sum_{x^{\prime }-\left\{x_{j}^{\prime },x_{k}^{\prime }\right\}}\left\{\left(\prod_{i\neq j,k}P_{x_{i},x_{i}^{\prime }}\left(0\right)\right)\sum_{{\hat{r}}_{j}^{\prime },{\hat{r}}_{k}^{\prime }}P\left({\hat{r}}_{j}^{\prime }\right)P\left({\hat{r}}_{k}^{\prime }\right)\left(R^{j,k}\left(x_{1},x^{\prime }\right)-R^{j,k}\left(x_{2},x^{\prime }\right)\right)\right\}. \end{array}$$

Since $x_{1,k}=x_{2,k}$, we have $P_{s_{1,k},s_{k}^{\prime }}\left(a,{\hat{r}}_{1,k}\right)=P_{s_{2,k},s_{k}^{\prime }}\left(a,{\hat{r}}_{2,k}\right)$ for any $s_{k}^{\prime }$. We recall that the transition probability is independent of $\hat{r}$ when a=0. Combining with the fact that $s_{1,j}=s_{2,j}$, we also have $P_{s_{1,j},s_{j}^{\prime }}\left(0,{\hat{r}}_{1,j}\right)=P_{s_{2,j},s_{j}^{\prime }}\left(0,{\hat{r}}_{2,j}\right)$ for any $s_{j}^{\prime }$. Combining together, we obtain

$$P_{s_{1,k},s_{k}^{\prime }}\left(1,{\hat{r}}_{1,k}\right)P_{s_{1,j},s_{j}^{\prime }}\left(0,{\hat{r}}_{1,j}\right)=P_{s_{2,k},s_{k}^{\prime }}\left(1,{\hat{r}}_{2,k}\right)P_{s_{2,j},s_{j}^{\prime }}\left(0,{\hat{r}}_{2,j}\right),$$

$$P_{s_{1,k},s_{k}^{\prime }}\left(0,{\hat{r}}_{1,k}\right)=P_{s_{2,k},s_{k}^{\prime }}\left(0,{\hat{r}}_{2,k}\right).$$

Leveraging the above two problems, we have

$$\begin{array}{cc} R^{j,k}\left(x_{1},x^{\prime }\right) & -R^{j,k}\left(x_{2},x^{\prime }\right)=\\ & \sum_{s_{j}^{\prime },s_{k}^{\prime }}\left[P_{s_{k},s_{k}^{\prime }}\left(0,{\hat{r}}_{k}\right)\left(P_{s_{1,j},s_{j}^{\prime }}\left(1,{\hat{r}}_{1,j}\right)-P_{s_{2,j},s_{j}^{\prime }}\left(1,{\hat{r}}_{2,j}\right)\right)V\left(x^{\prime }\right)\right]. \end{array}$$

Consequently, we obtain

$$\begin{array}{cc} \delta^{j,k}\left(x_{1}\right)- & \delta^{j,k}\left(x_{2}\right)=\\ & \sum_{x^{\prime }-\left\{x_{j}^{\prime }\right\}}\left\{\prod_{i\neq j}P_{x_{i},x_{i}^{\prime }}\left(0\right)\left[\sum_{{\hat{r}}_{j}^{\prime }}P\left({\hat{r}}_{j}^{\prime }\right)\sum_{s_{j}^{\prime }}\left(P_{s_{1,j},s_{j}^{\prime }}\left(1,1\right)-P_{s_{2,j},s_{j}^{\prime }}\left(1,0\right)\right)V\left(x^{\prime }\right)\right]\right\}. \end{array}$$

In the following, we characterize the sign of

$$R_{1}≜\sum_{s_{j}^{\prime }}\left(P_{s_{1,j},s_{j}^{\prime }}\left(1,1\right)-P_{s_{2,j},s_{j}^{\prime }}\left(1,0\right)\right)V\left(x^{\prime }\right).$$

As $s_{1,j}=s_{2,j}>0$, for any $x^{\prime }-\left\{s_{j}^{\prime }\right\}$, we have

$$R_{1}=\left(\left(1-\alpha_{j}\right)-\left(1-\beta_{j}\right)\right)V\left(x^{\prime };s_{j}^{\prime }=0\right)+\left(\alpha_{j}-\beta_{j}\right)V\left(x^{\prime };s_{j}^{\prime }=s_{1,j}+1\right)\leq 0.$$

The inequality follows from Lemma 1 and the fact that $\beta_{j}>\alpha_{j}$. Since $\delta^{j,k}\left(x_{1}\right)-\delta^{j,k}\left(x_{2}\right)$ is a linear combination of $R_{1}$’s with non-negative coefficients, we can conclude that $\delta^{j,k}\left(x_{1}\right)\leq \delta^{j,k}\left(x_{2}\right)$. Since ${\hat{r}}_{1,j}>{\hat{r}}_{2,j}$, we can see that $\delta^{j,k}\left(x\right)$ is non-increasing in ${\hat{r}}_{j}$.

In a very similar way, we can show that $\delta^{j,k}\left(x\right)$ is non-decreasing in ${\hat{r}}_{k}$. We recall that ${\hat{r}}_{i}$ will not affect the system dynamic if $a_{i}=0$. Consequently, we can conclude that $\delta^{j,k}\left(x\right)$ is independent of ${\hat{r}}_{i}$ for any i≠j,k.

Combining together, we prove the second property.

Property 3—$\delta^{j,k}\left(x\right)\leq 0$ if $s_{k}\;=0$. The equality holds when s j =0 or ${\hat{r}}_{j}\;=\;p_{e,j}^{0}\;=\;0$.

Since the probabilities are non-negative, it is sufficient to show that $R^{j,k}\left(x,x^{\prime }\right)$ satisfies Property 3 for any $x^{\prime }-\left\{s_{j}^{\prime },s_{k}^{\prime }\right\}$. More precisely, it is sufficient to show that $R^{j,k}\left(x,x^{\prime }\right)\leq 0$ for any $x^{\prime }-\left\{s_{j}^{\prime },s_{k}^{\prime }\right\}$ when $s_{k}=0$ and the equality holds when $s_{j}=0$ or ${\hat{r}}_{j}=p_{e,j}^{0}=0$. We recall that $P_{s_{k},s_{k}^{\prime }}\left(1,{\hat{r}}_{k}\right)=P_{s_{k},s_{k}^{\prime }}\left(0,{\hat{r}}_{k}\right)$ for any $s_{k}^{\prime }$ when $s_{k}=0$. Hence, for any $x^{\prime }-\left\{s_{j}^{\prime },s_{k}^{\prime }\right\}$, we have

$$R^{j,k}\left(x,x^{\prime }\right)=\sum_{s_{k}^{\prime }}\left[P_{s_{k},s_{k}^{\prime }}\left(0,{\hat{r}}_{k}\right)\sum_{s_{j}^{\prime }}\left(P_{s_{j},s_{j}^{\prime }}\left(1,{\hat{r}}_{j}\right)-P_{s_{j},s_{j}^{\prime }}\left(0,{\hat{r}}_{j}\right)\right)V\left(x^{\prime }\right)\right].$$

Then, we investigate the following quantity for any $x^{\prime }-\left\{s_{j}^{\prime }\right\}$

$$R_{2}≜\sum_{s_{j}^{\prime }}\left(P_{s_{j},s_{j}^{\prime }}\left(1,{\hat{r}}_{j}\right)-P_{x_{j},x_{j}^{\prime }}\left(0,{\hat{r}}_{j}\right)\right)V\left(x^{\prime }\right).$$

To this end, we distinguish between the following cases

- When $s_{j}=0$, we have $P_{s_{j},s_{j}^{\prime }}\left(1,{\hat{r}}_{j}\right)=P_{s_{j},s_{j}^{\prime }}\left(0,{\hat{r}}_{j}\right)$ for any $s_{j}^{\prime }$. Thus, we conclude that $R_{2}=0$ for any $x^{\prime }-\left\{s_{j}^{\prime }\right\}$. Consequently, $R^{j,k}\left(x,x^{\prime }\right)=0$ for any $x^{\prime }-\left\{s_{j}^{\prime },s_{k}^{\prime }\right\}$.
- When $s_{j}>0$ and ${\hat{r}}_{j}=1$, for any $x^{\prime }-\left\{s_{j}^{\prime }\right\}$, we have
  (A7) $$R_{2}=\left(\alpha_{j}-1+p_{j}\right)V\left(x^{\prime };s_{j}^{\prime }=s_{j}+1\right)+\left(1-\alpha_{j}-p_{j}\right)V\left(x^{\prime };s_{j}^{\prime }=0\right)\leq 0$$
  The inequality follows from Lemma 1 and the fact that $\alpha_{j}+p_{j}<1$. Thus, $R^{j,k}\left(x,x^{\prime }\right)\leq 0$ for any $x^{\prime }-\left\{s_{j}^{\prime },s_{k}^{\prime }\right\}$.
- When $s_{j}>0$ and ${\hat{r}}_{j}=0$, by replacing the $\alpha_{j}$ in (A7) with $\beta_{j}$, we can get the same result. In this case, the equality holds when $\beta_{j}+p_{j}=1$, or, equivalently, $p_{e,j}^{0}=0$.

Combined together, we can conclude that Property 3 is true.

Property 4—$\delta^{j,k}\left(x\right)$ is non-increasing in $s_{j}$ if $\Gamma_{j}^{{\hat{r}}_{j}}\;\leq \;\Gamma_{k}^{{\hat{r}}_{k}}$ and is non-decreasing in $s_{k}$ if $\Gamma_{j}^{{\hat{r}}_{j}}\;\geq \;\Gamma_{k}^{{\hat{r}}_{k}}$ when $s_{j},s_{k}\;>\;0$. We define $\Gamma_{i}^{1}\;≜\;\frac{\alpha_{i}}{1\;-p_{i}}$ and $\Gamma_{i}^{0}\;≜\;\frac{\beta_{i}}{1\;-\;p_{i}}$ for 1 ≤ i ≤ N.

Such as we did in the proof of Property 3, it is sufficient to show that $R^{j,k}\left(x,x^{\prime }\right)$ satisfies Property 4 for any $x^{\prime }-\left\{s_{j}^{\prime },s_{k}^{\prime }\right\}$. We recall that $R^{j,k}\left(x,x^{\prime }\right)$ depends on the values of ${\hat{r}}_{j}$ and ${\hat{r}}_{k}$. Therefore, we distinguish between the following cases

- In the case of ${\hat{r}}_{j}={\hat{r}}_{k}=1$ and $s_{j},s_{k}>0$, for any $x^{\prime }-\left\{s_{j}^{\prime },s_{k}^{\prime }\right\}$, (A5) can be written as
  $$\begin{array}{cc} R^{j,k}\left(x,x^{\prime }\right) & =\sum_{s_{j}^{\prime },s_{k}^{\prime }}\left[\left(P_{s_{k},s_{k}^{\prime }}\left(0,1\right)P_{s_{j},s_{j}^{\prime }}\left(1,1\right)-P_{s_{k},s_{k}^{\prime }}\left(1,1\right)P_{s_{j},s_{j}^{\prime }}\left(0,1\right)\right)V\left(x^{\prime }\right)\right]\\ & =\left(p_{k}\alpha_{j}-\left(1-p_{j}\right)\left(1-\alpha_{k}\right)\right)V\left(x^{\prime };s_{j}^{\prime }=s_{j}+1;s_{k}^{\prime }=0\right)\\ & \;\;\;+\left(\left(1-p_{k}\right)\left(1-\alpha_{j}\right)-p_{j}\alpha_{k}\right)V\left(x^{\prime };s_{j}^{\prime }=0;s_{k}^{\prime }=s_{k}+1\right)\\ & \;\;\;+\left(\left(1-p_{k}\right)\alpha_{j}-\left(1-p_{j}\right)\alpha_{k}\right)V\left(x^{\prime };s_{j}^{\prime }=s_{j}+1;s_{k}^{\prime }=s_{k}+1\right)\\ & \;\;\;+\left(p_{k}\left(1-\alpha_{j}\right)-p_{j}\left(1-\alpha_{k}\right)\right)V\left(x^{\prime };s_{j}^{\prime }=0;s_{k}^{\prime }=0\right). \end{array}$$
  As we can verify
  $$p_{k}\alpha_{j}-\left(1-p_{j}\right)\left(1-\alpha_{k}\right)<\frac{1}{2}\left(p_{k}+p_{j}-1\right)<0,$$
  $$\left(1-p_{k}\right)\left(1-\alpha_{j}\right)-p_{j}\alpha_{k}>\frac{1}{2}\left(1-p_{k}-p_{j}\right)>0.$$
  We define $\Gamma_{i}^{1}≜\frac{\alpha_{i}}{1-p_{i}}$ and $\Gamma_{i}^{0}≜\frac{\beta_{i}}{1-p_{i}}$ for 1≤i≤N. Then, we have
  $$\Gamma_{j}^{1}⋚\Gamma_{k}^{1}⟹\left(1-p_{k}\right)\alpha_{j}-\left(1-p_{j}\right)\alpha_{k}⋚0.$$
  Combining with Lemma 1, we can conclude that, for any $x^{\prime }-\left\{s_{j}^{\prime },s_{k}^{\prime }\right\}$, $R^{j,k}\left(x,x^{\prime }\right)$ is non-increasing in $s_{j}$ if $\Gamma_{j}^{1}\leq \Gamma_{k}^{1}$ and is non-decreasing in $s_{k}$ if $\Gamma_{j}^{1}\geq \Gamma_{k}^{1}$.
- In the case of ${\hat{r}}_{j}={\hat{r}}_{k}=0$ and $s_{j},s_{k}>0$, by replacing the α’s in the above case with β’s, we can conclude with the same result.
- In the case of ${\hat{r}}_{j}=1$, ${\hat{r}}_{k}=0$, and $s_{j},s_{k}>0$, for any $x^{\prime }-\left\{s_{j}^{\prime },s_{k}^{\prime }\right\}$, (A5) can be written as
  $$\begin{array}{cc} R^{j,k}\left(x,x^{\prime }\right) & =\sum_{s_{j}^{\prime },s_{k}^{\prime }}\left[\left(P_{s_{k},s_{k}^{\prime }}\left(0,0\right)P_{s_{j},s_{j}^{\prime }}\left(1,1\right)-P_{s_{k},s_{k}^{\prime }}\left(1,0\right)P_{s_{j},s_{j}^{\prime }}\left(0,1\right)\right)V\left(x^{\prime }\right)\right]\\ & =\left(p_{k}\alpha_{j}-\left(1-p_{j}\right)\left(1-\beta_{k}\right)\right)V\left(x^{\prime };s_{j}^{\prime }=s_{j}+1;s_{k}^{\prime }=0\right)\\ & \;\;\;+\left(\left(1-p_{k}\right)\left(1-\alpha_{j}\right)-p_{j}\beta_{k}\right)V\left(x^{\prime };s_{j}^{\prime }=0;s_{k}^{\prime }=s_{k}+1\right)\\ & \;\;\;+\left(\left(1-p_{k}\right)\alpha_{j}-\left(1-p_{j}\right)\beta_{k}\right)V\left(x^{\prime };s_{j}^{\prime }=s_{j}+1;s_{k}^{\prime }=s_{k}+1\right)\\ & \;\;\;+\left(p_{k}\left(1-\alpha_{j}\right)-p_{j}\left(1-\beta_{k}\right)\right)V\left(x^{\prime };s_{j}^{\prime }=0;s_{k}^{\prime }=0\right). \end{array}$$
  As we can verify
  $$p_{k}\alpha_{j}-\left(1-p_{j}\right)\left(1-\beta_{k}\right)<p_{k}\left(p_{j}-\frac{1}{2}\right)<0,$$
  $$\left(1-p_{k}\right)\left(1-\alpha_{j}\right)-p_{j}\beta_{k}>\left(1-p_{k}\right)\left(\frac{1}{2}-p_{j}\right)>0.$$
  At the same time
  $$\Gamma_{j}^{1}⋚\Gamma_{k}^{0}⟹\left(1-p_{k}\right)\alpha_{j}-\left(1-p_{j}\right)\beta_{k}⋚0.$$
  Combined with Lemma 1, we can conclude that, for any $x^{\prime }-\left\{s_{j}^{\prime },s_{k}^{\prime }\right\}$, $R^{j,k}\left(x,x^{\prime }\right)$ is non-increasing in $s_{j}$ if $\Gamma_{j}^{1}\leq \Gamma_{k}^{0}$ and is non-decreasing in $s_{k}$ if $\Gamma_{j}^{1}\geq \Gamma_{k}^{0}$.
- In the case of ${\hat{r}}_{j}=0$, ${\hat{r}}_{k}=1$, and $s_{j},s_{k}>0$, by swapping the α’s and β’s in the above case, we can conclude with the same result.

Combined together, we conclude that $R^{j,k}\left(x,x^{\prime }\right)$ satisfies Property 3 for any $x^{\prime }-\left\{s_{j}^{\prime },s_{k}^{\prime }\right\}$. Consequently, $\delta^{j,k}\left(x\right)$ is non-increasing in $s_{j}$ if $\Gamma_{j}^{{\hat{r}}_{j}}\leq \Gamma_{k}^{{\hat{r}}_{k}}$ and is non-decreasing in $s_{k}$ if $\Gamma_{j}^{{\hat{r}}_{j}}\geq \Gamma_{k}^{{\hat{r}}_{k}}$ when $s_{j},s_{k}>0$.

Property 5— $\delta^{j,k}\left(x\right)\;\leq \;0$ if $s_{j}\;\geq \;s_{k},\;{\hat{r}}_{j}\;\geq \;{\hat{r}}_{k}$ and users *j* and *k* are statistically identical.

According to Property 3, it is sufficient to consider the case where $s_{j},s_{k}>0$. We notice that the sign of $\delta^{j,k}\left(x\right)$ can be captured by the sign of the quantity $Q^{j,k}\left(x,x^{\prime }\right)≜\sum_{{\hat{r}}_{j}^{\prime },{\hat{r}}_{k}^{\prime }}$$P\left({\hat{r}}_{j}^{\prime }\right)P\left({\hat{r}}_{k}^{\prime }\right)R^{j,k}\left(x,x^{\prime }\right)$. Thus, we divide our discussion into the following cases.

- We first consider the case of $s_{j}\geq s_{k}>0$ and ${\hat{r}}_{j}={\hat{r}}_{k}=0$. Leveraging the definition of statistically identical, for any $x^{\prime }-\left\{x_{j}^{\prime },x_{k}^{\prime }\right\}$, we have
  $$\begin{array}{cc} Q^{j,k}\left(x,x^{\prime }\right)=\sum_{{\hat{r}}_{j}^{\prime },{\hat{r}}_{k}^{\prime }}P\left({\hat{r}}_{j}^{\prime }\right)P\left({\hat{r}}_{k}^{\prime }\right)\kappa_{1}( & V(x^{\prime };x_{j}^{\prime }=\left(0,{\hat{r}}_{j}^{\prime }\right);x_{k}^{\prime }=\left(s_{k}+1,{\hat{r}}_{k}^{\prime }\right))-\\ & V(x^{\prime };x_{j}^{\prime }=\left(s_{j}+1,{\hat{r}}_{j}^{\prime }\right);x_{k}^{\prime }=\left(0,{\hat{r}}_{k}^{\prime }\right))), \end{array}$$
  where $\kappa_{1}=1-p_{j}-\beta_{j}\geq 0$. Then, by substituting the values of $P(\hat{r})$ and using Lemma 2, we obtain   
  $$\begin{array}{cc} Q^{j,k}\left(x,x^{\prime }\right)= & \gamma_{j}\gamma_{k}\kappa_{1}V\left(x^{\prime };x_{j}^{\prime }=\left(s_{k}+1,1\right);x_{k}^{\prime }=\left(0,1\right)\right)-\\ & \gamma_{j}\gamma_{k}\kappa_{1}V\left(x^{\prime };x_{j}^{\prime }=\left(s_{j}+1,1\right);x_{k}^{\prime }=\left(0,1\right)\right)+\\ & \left(1-\gamma_{j}\right)\left(1-\gamma_{k}\right)\kappa_{1}V\left(x^{\prime };x_{j}^{\prime }=\left(s_{k}+1,0\right);x_{k}^{\prime }=\left(0,0\right)\right)-\\ & \left(1-\gamma_{j}\right)\left(1-\gamma_{k}\right)\kappa_{1}V\left(x^{\prime };x_{j}^{\prime }=\left(s_{j}+1,0\right);x_{k}^{\prime }=\left(0,0\right)\right)+\\ & \gamma_{k}\left(1-\gamma_{j}\right)\kappa_{1}V\left(x^{\prime };x_{j}^{\prime }=\left(s_{k}+1,1\right);x_{k}^{\prime }=\left(0,0\right)\right)-\\ & \gamma_{k}\left(1-\gamma_{j}\right)\kappa_{1}V\left(x^{\prime };x_{j}^{\prime }=\left(s_{j}+1,0\right);x_{k}^{\prime }=\left(0,1\right)\right)+\\ & \gamma_{j}\left(1-\gamma_{k}\right)\kappa_{1}V\left(x^{\prime };x_{j}^{\prime }=\left(s_{k}+1,0\right);x_{k}^{\prime }=\left(0,1\right)\right)-\\ & \gamma_{j}\left(1-\gamma_{k}\right)\kappa_{1}V\left(x^{\prime };x_{j}^{\prime }=\left(s_{j}+1,1\right);x_{k}^{\prime }=\left(0,0\right)\right). \end{array}$$
  Since users *j* and *k* are statistically identical, we have $\gamma_{j}=\gamma_{k}$. Then, by Lemma 1, we have $Q^{j,k}\left(x,x^{\prime }\right)\leq 0$ for any $x^{\prime }-\left\{x_{j}^{\prime },x_{k}^{\prime }\right\}$. Since $\delta^{j,k}\left(x\right)$ is a linear combination of $Q^{j,k}\left(x,x^{\prime }\right)$’s with non-negative coefficients, we can conclude that $\delta^{j,k}\left(x\right)\leq 0$.
- For the case of $s_{j}\geq s_{k}>0$ and ${\hat{r}}_{j}={\hat{r}}_{k}=1$, by replacing $\beta_{j}$ in $\kappa_{1}$ with $\alpha_{j}$, we can conclude with the same result.
- Then, we consider the case of $s_{j}\geq s_{k}>0$, ${\hat{r}}_{j}=1$, and ${\hat{r}}_{k}=0$. We first notice that, for any $x^{\prime }-\left\{s_{j}^{\prime },s_{k}^{\prime }\right\}$
  $$\begin{array}{cc} R^{j,k}\left(x,x^{\prime }\right)= & \left(p_{k}\alpha_{j}-\left(1-p_{j}\right)\left(1-\beta_{k}\right)\right)V\left(x^{\prime };s_{j}^{\prime }=s_{j}+1;s_{k}^{\prime }=0\right)+\\ & \left(\left(1-p_{k}\right)\left(1-\alpha_{j}\right)-p_{j}\beta_{k}\right)V\left(x^{\prime };s_{j}^{\prime }=0;s_{k}^{\prime }=s_{k}+1\right)+\\ & \left(\left(1-p_{k}\right)\alpha_{j}-\left(1-p_{j}\right)\beta_{k}\right)V\left(x^{\prime };s_{j}^{\prime }=s_{j}+1;s_{k}^{\prime }=s_{k}+1\right)+\\ & \left(p_{k}\left(1-\alpha_{j}\right)-p_{j}\left(1-\beta_{k}\right)\right)V\left(x^{\prime };s_{j}^{\prime }=0;s_{k}^{\prime }=0\right). \end{array}$$
  As users *j* and *k* are statistically identical, we have $p_{j}=p_{k}$ and $\alpha_{j}<\beta_{k}$. Leveraging Lemma 1, we have
  $$\begin{array}{cc} R^{j,k}(x,x^{\prime })\leq & \left(\alpha_{j}+p_{j}-1\right)(V\left(x^{\prime };s_{j}^{\prime }=s_{j}+1;s_{k}^{\prime }=0\right)-\\ & V(x^{\prime };s_{j}^{\prime }=0;s_{k}^{\prime }=s_{k}+1)). \end{array}$$
  Then, for any $x^{\prime }-\left\{x_{j}^{\prime },x_{k}^{\prime }\right\}$
  $$\begin{array}{cc} Q^{j,k}\left(x,x^{\prime }\right)\leq \sum_{{\hat{r}}_{j}^{\prime },{\hat{r}}_{k}^{\prime }}P\left({\hat{r}}_{j}^{\prime }\right)P\left({\hat{r}}_{k}^{\prime }\right)\kappa_{2} & (V(x^{\prime };x_{j}^{\prime }=\left(0,{\hat{r}}_{j}^{\prime }\right);x_{k}^{\prime }=\left(s_{k}+1,{\hat{r}}_{k}^{\prime }\right))-\\ & V(x^{\prime };x_{j}^{\prime }=\left(s_{j}+1,{\hat{r}}_{j}^{\prime }\right);x_{k}^{\prime }=\left(0,{\hat{r}}_{k}^{\prime }\right))), \end{array}$$
  where $\kappa_{2}=1-p_{j}-\alpha_{j}>0$. Such as we did in the previous cases, we can leverage Lemmas 1 and 2 to conclude that $Q^{j,k}\left(x,x^{\prime }\right)\leq 0$ for any $x^{\prime }-\left\{x_{j}^{\prime },x_{k}^{\prime }\right\}$. Consequently, $\delta^{j,k}\left(x\right)\leq 0$ in this case. The details are omitted for the sake of space.

Combined together, we conclude the proof of Property 5.

## Appendix D. Proof of Corollary 2

We follow the same steps as in the proof of Lemma 1. To prove the corollary, it is sufficient to show that $V\left(x_{1}\right)\leq V\left(x_{2}\right)$ when $s_{1}<s_{2}$ and ${\hat{r}}_{1}={\hat{r}}_{2}$. We use mathematical induction to prove the monotonicity. First of all, the base case (i.e., ν=0) is true by initialization. We assume the lemma holds at iteration ν. Then, we want to examine whether it holds at iteration ν+1. For the system with a single user, the update step reported in problem (5) can be simplified and rewritten as follows

(A8) $$V_{\nu +1}\left(x\right)=\min_{a\in \{0,1\}}V_{\nu +1}^{a}\left(x\right),$$

where

$$V_{\nu +1}^{a}\left(x\right)=C\left(x,a\right)-\theta +\sum_{{\hat{r}}^{\prime }}P\left({\hat{r}}^{\prime }\right)\sum_{s^{\prime }}P_{s,s^{\prime }}\left(a,\hat{r}\right)V_{\nu }\left(x^{\prime }\right),$$

and θ is the optimal value for $M_{1}\left(\lambda ,-1\right)$. To prove the desired results, we distinguish between the following cases

- We first consider the case of $s_{1}=0<s_{2}$ and ${\hat{r}}_{1}={\hat{r}}_{2}=0$. When a=1, we have
  $$V_{\nu +1}^{1}\left(x_{1}\right)=C\left(x_{1},1\right)-\theta +\sum_{{\hat{r}}^{\prime }}P\left({\hat{r}}^{\prime }\right)(pV_{\nu }\left(1,{\hat{r}}^{\prime }\right)+\left(1-p\right)V_{\nu }\left(0,{\hat{r}}^{\prime }\right)),$$
  $$\begin{array}{c} V_{\nu +1}^{1}\left(x_{2}\right)=C\left(x_{2},1\right)-\theta +\sum_{{\hat{r}}^{\prime }}P\left({\hat{r}}^{\prime }\right)(\beta V_{\nu }\left(s_{2}+1,{\hat{r}}^{\prime }\right)+\left(1-\beta \right)V_{\nu }\left(0,{\hat{r}}^{\prime }\right)). \end{array}$$
  Subtracting the two expressions yields
  $$\begin{array}{cc} V_{\nu +1}^{1}\left(x_{1}\right) & -\;V_{\nu +1}^{1}\left(x_{2}\right)\\ & \leq C\left(x_{1},1\right)-C\left(x_{2},1\right)+\sum_{{\hat{r}}^{\prime }}P\left({\hat{r}}^{\prime }\right)\left[\left(p-\beta \right)\left(V_{\nu }\left(1,{\hat{r}}^{\prime }\right)-V_{\nu }\left(0,{\hat{r}}^{\prime }\right)\right)\right]\;\leq \;0. \end{array}$$
  The inequalities hold since β>p, C(x,a) is non-decreasing in *s*, and Corollary 2 is true at iteration ν by assumption.
  For the case of a=0, we obtain
  $$V_{\nu +1}^{0}\left(x_{1}\right)=C\left(x_{1},0\right)-\theta +\sum_{{\hat{r}}^{\prime }}P\left({\hat{r}}^{\prime }\right)(pV_{\nu }\left(1,{\hat{r}}^{\prime }\right)+\left(1-p\right)V_{\nu }\left(0,{\hat{r}}^{\prime }\right)),$$
  $$\begin{array}{c} V_{\nu +1}^{0}\left(x_{2}\right)=C\left(x_{2},0\right)-\theta +\sum_{{\hat{r}}^{\prime }}P\left({\hat{r}}^{\prime }\right)(\left(1-p\right)V_{\nu }\left(s_{2}+1,{\hat{r}}^{\prime }\right)+pV_{\nu }\left(0,{\hat{r}}^{\prime }\right)). \end{array}$$
  Therefore, when a=0, we have
  $$\begin{array}{cc} V_{\nu +1}^{0}\left(x_{1}\right) & -V_{\nu +1}^{0}\left(x_{2}\right)\\ & \leq C\left(x_{1},0\right)-C\left(x_{2},0\right)+\sum_{{\hat{r}}^{\prime }}P\left({\hat{r}}^{\prime }\right)\left[\left(2p-1\right)\left(V_{\nu }\left(1,{\hat{r}}^{\prime }\right)-V_{\nu }\left(0,{\hat{r}}^{\prime }\right)\right)\right]\;\leq \;0. \end{array}$$
  The inequalities hold since 2p−1<0, C(x,a) is non-decreasing in *s*, and Corollary 2 is true at iteration ν by assumption. Combined together, we can see that $V_{\nu +1}^{a}\left(x_{1}\right)\leq V_{\nu +1}^{a}\left(x_{2}\right)$ for any feasible *a*. Then, by problem (A8), we can conclude that the lemma holds at iteration ν+1 when $s_{1}=0<s_{2}$ and ${\hat{r}}_{1}={\hat{r}}_{2}=0$.
- When $s_{1}=0<s_{2}$ and ${\hat{r}}_{1}={\hat{r}}_{2}=1$, by replacing the β’s in the above case with α’s, we can achieve the same result.
- When $0<s_{1}<s_{2}$ and ${\hat{r}}_{1}={\hat{r}}_{2}$, we notice that $P_{s_{1},s_{1}+1}\left(a,{\hat{r}}_{1}\right)=P_{s_{2},s_{2}+1}\left(a,{\hat{r}}_{2}\right)$ and $P_{s_{1},0}\left(a,{\hat{r}}_{1}\right)=P_{s_{2},0}\left(a,{\hat{r}}_{2}\right)$. Then, leveraging the monotonicity of $V_{\nu }\left(x\right)$ and C(x,a), we can conclude with the same result.

Combining the three cases, we prove that the lemma holds at iteration ν+1 of VIA. Therefore, the lemma holds at any iteration ν by mathematical induction. Since VIA is guaranteed to converge to the value function when ν→+∞, we can conclude our proof.

## Appendix E. Proof of Proposition 1

We define $\Delta V\left(x\right)≜V^{1}\left(x\right)-V^{0}\left(x\right)$ where $V^{a}\left(x\right)$ is the value function resulting from taking action *a* at state *x*. Then, $V^{a}\left(x\right)$ can be calculated as follows

(A9) $$V^{a}\left(x\right)=C\left(x,a\right)-\theta +\sum_{x^{\prime }\in X}P_{x,x^{\prime }}\left(a\right)V\left(x^{\prime }\right),$$

where θ is the optimal value for $M_{1}\left(\lambda ,-1\right)$. Hence, the optimal action at state *x* can be fully characterized by the sign of ΔV(x). More precisely, the optimal action at state *x* is a=1 if ΔV(x)<0, and a=0 is optimal otherwise. To determine the sign of ΔV(x) for each state, we distinguish between the following cases

- We first consider the state $x=(0,\hat{r})$. Applying the results in Section 2.3 to problem (A9), we obtain
  $$\begin{array}{cc} V^{0}\left(0,\hat{r}\right)= & -\theta +(1-\gamma )(1-p)V(0,0)+(1-\gamma )pV(1,0)+\\ & \gamma (1-p)V(0,1)+\gamma pV(1,1), \end{array}$$
  (A10) $$V^{1}\left(0,\hat{r}\right)=\lambda +V^{0}\left(0,\hat{r}\right).$$
  Therefore, $\Delta V(0,\hat{r})=\lambda \geq 0$. Thus, the optimal action at state $\left(0,\hat{r}\right)$ is a=0.
- Then, we consider the state x=(s,0) where s>0. Applying the results in Section 2.3 to Equation (A9), we obtain
  $$\begin{array}{cc} V^{0}\left(s,0\right) & =f(s)-\theta +(1-\gamma )pV(0,0)+(1-\gamma )(1-p)V(s+1,0)+\\ & \;\;\;\;\gamma pV(0,1)+\gamma (1-p)V(s+1,1), \end{array}$$
  $$\begin{array}{cc} V^{1}\left(s,0\right) & =f(s)+\lambda -\theta +(1-\gamma )(1-\beta )V(0,0)+(1-\gamma )\beta V(s+1,0)+\\ & \;\;\;\;\gamma (1-\beta )V(0,1)+\gamma \beta V(s+1,1). \end{array}$$
  Then,
  (A11) $$\Delta V\left(s,0\right)=\lambda +p_{e}^{0}\left(1-2p\right)\omega ,$$
  where ω=(1−γ)[V(0,0)−V(s+1,0)]+γ[V(0,1)−V(s+1,1)]≤0.
- Finally, we consider the state x=(s,1) where s>0. Following the same trajectory, we have
  $$\begin{array}{c} \Delta V\left(s,1\right)=\lambda +\left(1-p_{e}^{1}\right)\left(1-2p\right)\omega . \end{array}$$

According to Corollary 2 and the fact that p<0.5, we can see that ΔV(s,0) and ΔV(s,1) are both a constant λ plus a term that is non-increasing in *s*. As the time penalty function is unbounded, the value function must also be unbounded. Then, combining the three cases, we can conclude the following. For fixed $\hat{r}$, there always exists a threshold $n_{\hat{r}}>0$ such that the optimal action at state $\left(s,\hat{r}\right)$ where $s\geq n_{\hat{r}}$ is a=1, otherwise a=0 is optimal. Since $\hat{r}\in \left\{0,1\right\}$, the optimal policy can be fully captured by the pair $\left(n_{0},n_{1}\right)$.

In the following, we determine the relationship between $n_{0}$ and $n_{1}$. We have

$$\Delta V\left(s,1\right)-\Delta V\left(s,0\right)=\left(1-p_{e}^{1}-p_{e}^{0}\right)\left(1-2p\right)\omega \leq 0.$$

At the same time, for the threshold $n_{0}$, we know $\Delta V(n_{0},0)<0$. Then, we have $\Delta V\left(n_{0},1\right)\leq \Delta V\left(n_{0},0\right)<0$. Combined with the fact that $\Delta V(s,\hat{r})$ is non-increasing in *s*, we can conclude that the ordering $n_{0}\geq n_{1}$ is true.

## Appendix F. Proof of Proposition 2

We notice that the dynamic of AoII under threshold policy can be fully captured by a Discrete-Time Markov Chain (DTMC). Then, the expected AoII ${\bar{\Delta }}_{n}$ and the expected transmission rate ${\bar{\rho }}_{n}$ under threshold policy $n=(n_{0},n_{1})$ can be obtained from the stationary distribution of the induced DTMC. Let the states of the induced DTMC be the values of *s*. We recall that $\hat{r}$ is an independent Bernoulli random variable with parameter γ. Combined with the results in Section 2.3, we can easily obtain the state transition probabilities of the induced DTMC, which are shown in Figure A1.

The balance equations of the induced DTMC are the following

$$\left(1-p\right)\pi_{0}+p\sum_{k=1}^{n_{1}-1}\pi_{k}+\left(1-c_{1}\right)\sum_{k=n_{1}}^{n_{0}-1}\pi_{k}+\left(1-c_{2}\right)\sum_{k=n_{0}}^{+\infty }\pi_{k}=\pi_{0}.$$

$$p\pi_{0}=\pi_{1}.$$

$$\left(1-p\right)\pi_{k-1}=\pi_{k}\;\;for\;2\leq k\leq n_{1}.$$

$$c_{1}\pi_{k-1}=\pi_{k}\;\;for\;n_{1}+1\leq k\leq n_{0}.$$

$$c_{2}\pi_{k-1}=\pi_{k}\;\;for\;n_{0}+1\leq k.$$

$$\sum_{k=0}^{+\infty }\pi_{k}=1.$$

Then, we can easily solve the above system of linear equations. After some algebraic manipulation, we obtain the following

$$\pi_{0}=\frac{1}{2+p{\left(1-p\right)}^{n_{1}-1}\left[\frac{1}{1-c_{1}}-\frac{1}{p}+c_{1}^{n_{0}-n_{1}}\left(\frac{1}{1-c_{2}}-\frac{1}{1-c_{1}}\right)\right]}.$$

$$\pi_{k}=p{\left(1-p\right)}^{k-1}\pi_{0}\;\;for\;1\leq k\leq n_{1}.$$

$$\pi_{k}=p{\left(1-p\right)}^{n_{1}-1}c_{1}^{k-n_{1}}\pi_{0}\;\;for\;n_{1}+1\leq k\leq n_{0}.$$

$$\pi_{k}=p{\left(1-p\right)}^{n_{1}-1}c_{1}^{n_{0}-n_{1}}c_{2}^{k-n_{0}}\pi_{0}\;\;for\;n_{0}+1\leq k.$$

Equipped with the above results, we proceed with calculating ${\bar{\Delta }}_{n}$ and ${\bar{\rho }}_{n}$. According to problem (6a), the expected AoII is:

$${\bar{\Delta }}_{n}=\sum_{k=0}^{+\infty }f\left(k\right)\pi_{k}.$$

Substituting the expressions of $\pi_{k}$’s, we can get the expression of ${\bar{\Delta }}_{n}$. Proposition 1 tells us the following.

- For state $\left(s,\hat{r}\right)$ where $s<n_{1}$, it is optimal to stay idle (i.e., a=0).
- For state $\left(s,\hat{r}\right)$ where $n_{1}\leq s<n_{0}$, it is optimal to make a transmission attempt only when $\hat{r}=1$. We recall that $\hat{r}$ is an independent Bernoulli random variable with parameter γ. Therefore, the expected proportion of time that the system is at state (s,1) is $\gamma \pi_{s}$.
- For state $\left(s,\hat{r}\right)$ where $s\geq n_{0}$, it is optimal to make transmission attempt regardless of $\hat{r}$.

Combined with problem (6b), we have

$${\bar{\rho }}_{n}=\gamma \sum_{k=n_{1}}^{n_{0}-1}\pi_{k}+\sum_{k=n_{0}}^{+\infty }\pi_{k}.$$

Substituting the expressions of $\pi_{k}$’s, we can obtain the closed-form expression of ${\bar{\rho }}_{n}$.

## Appendix G. Proof of Proposition 4

We first tackle the Whittle’s indexes at state $\left(0,\hat{r}\right)$ and (s,0) where s>0. To this end, we distinguish between the following cases

- We first consider the state $x=(0,\hat{r})$. By definition, Whittle’s index is the infimum λ such that $V^{0}\left(x\right)=V^{1}\left(x\right)$. According to (A10), we can conclude that $W_{x}=0$ when $x=(0,\hat{r})$.
- Then, we consider the state x=(s,0) where s>0. We recall that $p_{e}^{0}=0$. Then, we can conclude, from (A11), that $W_{x}=0$ for all x=(s,0) where s>0.

Now, we tackle the Whittle’s index at state x=(s,1) where s>0. For convenience, we denote by $W_{n}$ the Whittle’s index at state x=(n,1). According to the monotonicity of ΔV(x) shown in the proof of Proposition 1, we can conclude that threshold policy n=(+∞,n+1) is optimal when $V^{0}\left(n,1\right)=V^{1}\left(n,1\right)$. Then, we can prove the following

**Lemma** **A1.**
*When (9) is satisfied and $V^{0}\left(n,1\right)=V^{1}\left(n,1\right)$, V(s,1)=V(s,0)≜V(s) for 0≤s≤n.*

**Proof.** Since the value function satisfies the Bellman equation, it is sufficient to show that V(s,1) and V(s,0) satisfy the same Bellman equation. We recall that the Bellman equation for V(x) is given by

$$V\left(x\right)=\min_{a\in \{0,1\}}V^{a}\left(x\right),$$

where

(A12) $$V^{a}\left(x\right)=C\left(x,a\right)-\theta +\sum_{x^{\prime }}P_{x,x^{\prime }}\left(a\right)V\left(x^{\prime }\right),$$

and θ is the optimal value of the decoupled problem. We recall, from Corollary 3, that the optimal action at state (s,0) is staying idle (i.e., a=0) for any *s*. We also know that threshold policy n=(+∞,n+1) is optimal when $V^{0}\left(n,1\right)=V^{1}\left(n,1\right)$. Therefore, the optimal actions at states (s,0) and (s,1) where s≤n are the same (i.e., a=0). Equivalently, we have

(A13) $$V\left(s,\hat{r}\right)=V^{0}\left(s,\hat{r}\right),\;for\;s\leq n.$$

According to the system dynamic reported in Section 2.3, we know that the state transition probabilities are independent of $\hat{r}$ when a=0. Meanwhile, $\hat{r}$ does not affect the instant cost. Let $x_{1}=\left(s,1\right)$ and $x_{2}=\left(s,0\right)$. Then, for any $x^{\prime }$, we have

$$P_{x_{1},x^{\prime }}\left(0\right)=P_{x_{2},x^{\prime }}\left(0\right).$$

$$C\left(x_{1},0\right)=C\left(x_{2},0\right).$$

Hence, according to (A12), we can see that $V^{0}\left(s,0\right)=V^{0}\left(s,1\right)$ for any s≤n. Combined with problem (A13), we can conclude that V(s,0)=V(s,1) for any 0≤s≤n.    ☐

By definition, Whittle’s index $W_{n}$ is the infimum λ such that $V^{0}\left(n,1\right)=V^{1}\left(n,1\right)$. In this case, according to Lemma A1, V(0,1)=V(0,0)=V(0). Then, $V^{0}\left(n,1\right)$ and $V^{1}\left(n,1\right)$ can be written as

(A14) $$V^{0}\left(n,1\right)=f\left(n\right)-\theta +pV\left(0\right)+\left(1-p\right)\left[\left(1-\gamma \right)V\left(n+1,0\right)+\gamma V\left(n+1,1\right)\right].$$

$$\begin{array}{cc} V^{1}\left(n,1\right) & =f\left(n\right)+W_{n}-\theta +\left(1-\alpha \right)V\left(0\right)+\alpha \left[\left(1-\gamma \right)V\left(n+1,0\right)+\gamma V\left(n+1,1\right)\right]. \end{array}$$

Without a loss of generality, we assume V(0)=0. Then, equating the two expressions yields

(A15) $$W_{n}=\left(1-p-\alpha \right)\left(\gamma V\left(n+1,1\right)+\left(1-\gamma \right)V\left(n+1,0\right)\right).$$

Combining problems (A14) and (A15), we conclude that $W_{n}$ is

$$W_{n}=\frac{\left(1-p-\alpha \right)(V^{0}\left(n,1\right)+\theta -f\left(n\right))}{1-p}.$$

Since the optimal action at state (n,1) is a=0, we have $V^{0}\left(n,1\right)=V\left(n,1\right)=V\left(n\right)$. Finally, we obtain

(A16) $$W_{n}=\frac{\left(1-p-\alpha )(V(n)+\theta -f(n)\right)}{1-p}.$$

Now, we tackle the expression of V(n). When $V^{0}\left(n,1\right)=V^{1}\left(n,1\right)$, the optimal action at state $\left(s,\hat{r}\right)$ where 0≤s<n is staying idle. Then, leveraging Lemma A1, value function V(s) where 0≤s<n satisfies the following

(A17) $$V\left(s\right)=\left\{\begin{array}{cc} -\theta +f(0)+pV(1) & when\;s=0,\\ -\theta +f(s)+(1-p)V(s+1) & when\;0<s<n. \end{array}\right.$$

By backward induction, we end up with the following equation for 0<s<n.

$$V\left(s\right)=\frac{-\theta (1-{\left(1-p\right)}^{n-s})}{p}+\sum_{k=1}^{n-s}f\left(n-k\right){\left(1-p\right)}^{n-s-k}+{\left(1-p\right)}^{n-s}V\left(n\right).$$

Letting s=1 yields

$$V\left(1\right)=\frac{-\theta (1-{\left(1-p\right)}^{n-1})}{p}+\sum_{k=1}^{n-1}f\left(n-k\right){\left(1-p\right)}^{n-1-k}+{\left(1-p\right)}^{n-1}V\left(n\right).$$

From problem (A17), V(1) also satisfies the following

$$V\left(1\right)=\frac{\theta -f(0)}{p}.$$

Equating the two expressions of V(1), we obtain

(A18) $$V\left(n\right)=\frac{-f(0)}{p{\left(1-p\right)}^{n-1}}+\theta \left(\frac{2}{p{\left(1-p\right)}^{n-1}}-\frac{1}{p}\right)-\sum_{k=1}^{n-1}f\left(n-k\right){\left(1-p\right)}^{-k}.$$

We recall that, when $V^{0}\left(n,1\right)=V^{1}\left(n,1\right)$, threshold policy n=(+∞,n+1) is optimal and both actions at state x=(n,1) are equally desirable. Thus, threshold policy n=(+∞,n) is also optimal. Then, we know

(A19) $$\theta ={\bar{\Delta }}_{n}+W_{n}{\bar{\rho }}_{n},$$

where ${\bar{\Delta }}_{n}$ and ${\bar{\rho }}_{n}$ are the expected AoII and the expected transmission rate under threshold policy n=(+∞,n), respectively. Finally, combining problems (A16), (A18) and (A19), we obtain

$$W_{n}=\frac{\frac{-f(0)}{p{\left(1-p\right)}^{n}}+{\bar{\Delta }}_{n}\frac{2-{\left(1-p\right)}^{n}}{p{\left(1-p\right)}^{n}}-{\left(1-p\right)}^{-n}\left(\sum_{k=1}^{n}f\left(k\right){\left(1-p\right)}^{k-1}\right)}{\frac{1}{1-p-\alpha }-{\bar{\rho }}_{n}\frac{2-{\left(1-p\right)}^{n}}{p{\left(1-p\right)}^{n}}}.$$

After some algebraic manipulation, we have

$$W_{n}=\frac{\left(1-c_{1}\right)\sum_{k=n+1}^{+\infty }f\left(k\right)c_{1}^{k-n-1}-{\bar{\Delta }}_{n}}{\frac{\left(1-c_{1}\right)\left(1-p\right)-\gamma \left(1-p-\alpha \right)}{c_{1}\left(1-p-\alpha \right)}+{\bar{\rho }}_{n}},$$

where $c_{1}=\left(1-\gamma \right)\left(1-p\right)+\gamma \alpha$.

In the following, we investigate some properties of Whittle’s index. First of all, $W_{n}$ is non-negative since 1−p−α and $V(n+1,\hat{r})$ in (A15) are all non-negative. Meanwhile, combining (A15) with the fact that $V(n,\hat{r})$ is non-decreasing in *n*, we can verify that $W_{n}$ is non-decreasing in *n*. Combined with the Whittle’s indexes in two other cases (i.e., $x=(0,\hat{r})$ and x=(s,0) where s>0), we can easily obtain the properties of $W_{x}$ as detailed in Proposition 4.

## Appendix H. Proof of Proposition 5

We notice that $M_{1}\left(\lambda ,-1\right)$ coincides with the decoupled model studied in Section 4.2. When problem (9) is satisfied, the decoupled problem is indexable, and, according to Corollary 3, we only need to show that *n* is the optimal threshold for the states with $\hat{r}=1$. We first tackle the case of λ>0. To this end, we divide our discussion into the following cases

- For state (s,1) where s<n, $W_{s}\leq \lambda$ by definition. As the problem is indexable, we have $D\left(W_{s}\right)\subseteq D\left(\lambda \right)$. We recall that $W_{s}≜\min\left\{\lambda^{\prime }\geq 0:V^{0}\left(s,1\right)=V^{1}\left(s,1\right)\right\}$. Equivalently, $W_{s}≜\min\left\{\lambda^{\prime }\geq 0:\left(s,1\right)\in D\left(\lambda^{\prime }\right)\right\}$. Then, we know $\left(s,1\right)\in D\left(W_{s}\right)$. Combined together, we conclude that (s,1)∈D(λ). In other words, the optimal action at state (s,1) where s<n is to stay idle (i.e., a=0).
- For state (s,1) where s≥n, we first recall that $W_{s}=\min\left\{\lambda^{\prime }\geq 0:\left(s,1\right)\in D\left(\lambda^{\prime }\right)\right\}$. Consequently, for any $\lambda^{\prime }<W_{s}$, we know $\left(s,1\right)\notin D\left(\lambda^{\prime }\right)$. Meanwhile, we have $W_{s}\geq W_{n}>\lambda$ by the monotonicity of Whittle’s index and the definition of *n*. Hence, we can conclude that (s,1)∉D(λ). In other words, the optimal action at state (s,1) where s≥n is to make the transmission attempt.

Then, we conclude that *n* is the optimal threshold for the states with $\hat{r}=1$ when λ>0. In the case of λ=0, according to the proof of Proposition 1, we can easily verify that the optimal threshold is 1.

## Appendix I. Proof of Theorem 2

We first make the following definitions. When $M_{1}\left(\lambda ,-1\right)$ is at state *x* and action *a* is taken, cost $C_{1}\left(x,a\right)≜f\left(s\right)$ and $C_{2}\left(x,a\right)≜\lambda a$ are incurred. We denote the expected $C_{1}$-cost and the expected $C_{2}$-cost under policy ϕ as ${\bar{C}}_{1}\left(\phi \right)$ and ${\bar{C}}_{2}\left(\phi \right)$, respectively. Let *G* be a non-empty set of states. For the given state *i*, we define $R^{*}\left(i,G\right)$ as the class of policies ϕ, for which the following hold

- The probability $P_{\phi }\left(x_{n}\in G\;for\;some\;n\geq 1\;|\;x_{0}=i\right)=1$ where $x_{n}$ is the state of $M_{1}\left(\lambda ,-1\right)$ at time *n*.
- The expected time $m_{iG}\left(\phi \right)$ of a first passage from *i* to *G* under ϕ is finite.
- The expected $C_{1}$-cost ${\bar{C}}_{1}^{i,G}\left(\phi \right)$ and the expected $C_{2}$-cost ${\bar{C}}_{2}^{i,G}\left(\phi \right)$ of a first passage form *i* to *G* under ϕ are finite.

With the definitions in mind, we proceed with verifying the assumptions given in [27].

1. *For all d>0, the set A(d)={x| there exists an action a such that $C_{1}\left(x,a\right)+C_{2}\left(x,a\right)\leq d\}$ is finite*: For any state *x*, the cost satisfies $C_{1}\left(x,a\right)+C_{2}\left(x,a\right)=f\left(s\right)+\lambda a\geq f\left(s\right)$. The equality holds when a=0. Then, the states in A(d) must satisfy f(s)≤d. Combined with the fact that f(s) is a non-decreasing and unbounded function when $s\in N_{0}$, we can conclude that A(d) is finite.
2. *There exists a stationary policy e such that the induced Markov chain has the following properties: the state space S consists of a single (non-empty) positive recurrent class R and a set U of transient states such that $e\in R^{*}\left(i,R\right)$ for i∈U. Moreover, both ${\bar{C}}_{1}\left(e\right)$ and ${\bar{C}}_{2}\left(e\right)$ on R are finite*: We consider the policy under which the base station makes a transmission attempt at every time slot. According to the system dynamic detailed in Section 2.3, we can see that all the states communicate with state (0,0) and (0,0) communicates with all other states. Thus, the state space S consists of a single (non-empty) positive recurrent class and the set of transient states can simply be an empty set. ${\bar{C}}_{1}\left(e\right)$ and ${\bar{C}}_{2}\left(e\right)$ are trivially finite as we can verify using Proposition 2.
3. *Given any two state x≠y, there exists a policy ϕ such that $\phi \in R^{*}\left(x,y\right)$*: We notice that, under any policy, the maximum increase of *s* between two consecutive time slots is 1. Meanwhile, when *s* decreases, it decreases to zero. Combined with the fact that $\hat{r}$ is an independent Bernoulli random variable, we can conclude that there always exists a path between any *x* and *y* with positive probability. $m_{xy}\left(\phi \right)$, ${\bar{C}}_{1}^{x,y}\left(\phi \right)$, and ${\bar{C}}_{2}^{x,y}\left(\phi \right)$ are trivially finite.
4. *If a stationary policy ϕ has at least one positive recurrent state, then it has a single positive recurrent class R. Moreover, if x=(0,0)∉R, then $\phi \in R^{*}\left(x,R\right)$*: Given that $\hat{r}$ is an independent Bernoulli random variable, we can easily conclude from the system dynamic that all the states communicate with state (0,0) and (0,0) communicates with all other states under any stationary policy. Therefore, any positive recurrent class must contain state (0,0). Thus, there must have only one positive recurrent class which is R=S.
5. *There exists a policy ϕ such that ${\bar{C}}_{1}\left(\phi \right)<\infty$ and ${\bar{C}}_{2}\left(\phi \right)<K$ where K∈(0,1]*: We notice that ${\bar{C}}_{1}\left(\phi \right)$ and ${\bar{C}}_{2}\left(\phi \right)$ are nothing but the expected AoII and the expected transmission rate achieved by ϕ, respectively. Then, we can easily verify that such policy exists using Proposition 2.

As the assumptions are verified, we proceed with introducing the optimal randomized policy for given λ. We say a policy is λ-optimal if the policy is optimal for $M_{1}\left(\lambda ,-1\right)$. We consider two monotone sequences $\lambda_{+}^{n}↓\lambda$ and $\lambda_{-}^{n}↑\lambda$. Then, there exist subsequences of $\lambda_{+}^{n}$ and $\lambda_{-}^{n}$ such that the corresponding sequences of optimal policies converge. Then, according to Lemma 3.7 of [27], the limit points, denoted by $n_{\lambda_{+}}$ and $n_{\lambda_{-}}$, are both λ-optimal. By Proposition 3.2 of [27], the Markov chains induced by $n_{\lambda_{+}}$ and $n_{\lambda_{-}}$ both contain a single non-empty positive recurrent class and state (0,0) is positive recurrent in both induced Markov chains. Hence, the base station can choose which policy to follow each time the system reaches state (0,0) while keeping the resulting randomized policy λ-optimal as suggested by Lemma 3.9 of [27]. More precisely, we consider the following randomized policy: each time the system reaches state (0,0), the base station will choose $n_{\lambda_{-}}$ with probability μ and $n_{\lambda_{+}}$ with probability 1−μ. The chosen policy will be followed until the next choice. We denote such policy as $n_{\lambda }$ and conclude that $n_{\lambda }$ is λ-optimal under any μ∈[0,1].

## Appendix J. Proof of Proposition 6

The value function V(x) and $V^{i}\left(x_{i}\right)$ must satisfy their own Bellman equations. More precisely

$$V\left(x\right)+\theta =\min_{a\in A_{N}\left(-1\right)}\left\{C\left(x,a\right)+\sum_{x^{\prime }}Pr\left(x^{\prime }|x,a\right)V\left(x^{\prime }\right)\right\},$$

(A20) $$V^{i}\left(x_{i}\right)+\theta_{i}=\min_{a_{i}\in \left\{0,1\right\}}\left\{C\left(x_{i},a_{i}\right)+\sum_{x_{i}^{\prime }}Pr\left(x_{i}^{\prime }|x_{i},a_{i}\right)V^{i}\left(x_{i}^{\prime }\right)\right\},$$

where θ and $\theta_{i}$ are the optimal values of $M_{N}\left(\lambda ,-1\right)$ and $M_{1}^{i}\left(\lambda ,-1\right)$, respectively. We recall from Section 2.3 that the users are independent when action a and current state x are given. Thus

$$Pr\left(x^{\prime }|x,a\right)=\prod_{i=1}^{N}Pr\left(x_{i}^{\prime }|x,a\right),$$

where $x^{\prime }=\left(x_{1}^{\prime },\ldots ,x_{N}^{\prime }\right)$. Then, we have

$$\sum_{x^{\prime }-\left\{x_{i}^{\prime }\right\}}Pr\left(x^{\prime }-\left\{x_{i}^{\prime }\right\}|x,a\right)=\sum_{x^{\prime }-\left\{x_{i}^{\prime }\right\}}\prod_{j\neq i}Pr\left(x_{j}^{\prime }|x,a\right)=1.$$

We also recall from Section 2.3 that the state of user *i* depends only on its previous state and the action with respect to user *i*. Thus

$$Pr\left(x_{i}^{\prime }|x,a\right)=Pr\left(x_{i}^{\prime }|x_{i},a_{i}\right).$$

Combined together, we obtain

(A21) $$\begin{array}{cc} \sum_{i=1}^{N}\sum_{x_{i}^{\prime }}Pr\left(x_{i}^{\prime }|x_{i},a_{i}\right)V^{i}\left(x_{i}^{\prime }\right) & =\sum_{i=1}^{N}\sum_{x_{i}^{\prime }}\left[\sum_{x^{\prime }-\left\{x_{i}^{\prime }\right\}}\prod_{j\neq i}Pr\left(x_{j}^{\prime }|x,a\right)\right]Pr\left(x_{i}^{\prime }|x_{i},a_{i}\right)V^{i}\left(x_{i}^{\prime }\right)\\ \; & =\sum_{i=1}^{N}\sum_{x_{i}^{\prime }}\left(\sum_{x^{\prime }-\left\{x_{i}^{\prime }\right\}}\prod_{i=1}^{N}Pr\left(x_{i}^{\prime }|x,a\right)V^{i}\left(x_{i}^{\prime }\right)\right)\\ \; & =\sum_{x^{\prime }}Pr\left(x^{\prime }|x,a\right)\left(\sum_{i=1}^{N}V^{i}\left(x_{i}^{\prime }\right)\right). \end{array}$$

Then, we sum problem (A20) over all users which yields

$$\sum_{i=1}^{N}\left(V^{i}\left(x_{i}\right)+\theta_{i}\right)=\min_{a}\left\{\sum_{i=1}^{N}\left(C\left(x_{i},a_{i}\right)+\sum_{x_{i}^{\prime }}Pr\left(x_{i}^{\prime }|x_{i},a_{i}\right)V^{i}\left(x_{i}^{\prime }\right)\right)\right\}.$$

We recall that $C\left(x,a\right)=\sum_{i=1}^{N}C\left(x_{i},a_{i}\right)$ by definition. Then, leveraging problem (A21), we obtain

$$\sum_{i=1}^{N}V^{i}\left(x_{i}\right)+\sum_{i=1}^{N}\theta_{i}=\min_{a\in A_{N}\left(-1\right)}\left\{C\left(x,a\right)+\sum_{x^{\prime }}Pr\left(x^{\prime }|x,a\right)\left(\sum_{i=1}^{N}V^{i}\left(x_{i}^{\prime }\right)\right)\right\}.$$

Since the solution to the Bellman equation is unique [21], we must have $\sum_{i=1}^{N}V^{i}\left(x_{i}\right)=V\left(x\right)$ and $\sum_{i=1}^{N}\theta_{i}=\theta$. Then, we can conclude that it is optimal for $M_{N}\left(\lambda ,-1\right)$ if each user adopts its own optimal policy.

## Appendix K. Proof of Theorem 3

In this proof, we class a policy as $\lambda^{*}$-optimal if it is optimal for $M_{N}\left(\lambda^{*},-1\right)$. In Section 4.2, we ensure that, for each user, there exists at least one threshold policy that yields a finite expected AoII. Therefore, we can conclude that, for RP, there exists at least one policy that causes the expected AoII and the expected transmission rate to be both finite. Then, according to Lemma 3.10 of [27], a policy is optimal for RP if

1. It is $\lambda^{*}$-optimal;
2. The resulting expected transmission rate is equal to *M*.

We first construct a policy $\phi_{\lambda^{*}}$ that is $\lambda^{*}$-optimal. We recall from Proposition 6 that a policy is $\lambda^{*}$-optimal if it consists of the optimal policies for each $M_{1}^{i}\left(\lambda^{*},-1\right)$ where 1≤i≤N. According to Theorem 2, for any *i*, there exist $n_{\lambda_{-}^{*},i}$ and $n_{\lambda_{+}^{*},i}$ that are both optimal for $M_{1}^{i}\left(\lambda^{*},-1\right)$. Then, we can construct the policy $\phi_{\lambda^{*}}$ in the following way.

- For user *i* with $n_{\lambda_{-}^{*},i}=n_{\lambda_{+}^{*},i}≜n_{\lambda^{*},i}$, the threshold policy $n_{\lambda^{*},i}$ is used. Then, the deterministic policy $n_{\lambda^{*},i}$ is optimal for $M_{1}^{i}\left(\lambda^{*},-1\right)$ and
  $${\bar{\rho }}^{i}\left(\lambda^{*}\right)={\bar{\rho }}^{i}\left(\lambda_{-}^{*}\right)={\bar{\rho }}^{i}\left(\lambda_{+}^{*}\right).$$
  In this case, the choice of $\mu_{i}$ makes no difference.
- For user *i* with $n_{\lambda_{-}^{*},i}\neq n_{\lambda_{+}^{*},i}$, the randomized policy $n_{\lambda^{*},i}$ as detailed in Theorem 2 is used. Then, for any $\mu_{i}\in \left[0,1\right]$, the randomized policy $n_{\lambda^{*},i}$ is optimal for $M_{1}^{i}\left(\lambda^{*},-1\right)$ and
  $${\bar{\rho }}^{i}\left(\lambda^{*}\right)=\mu_{i}{\bar{\rho }}^{i}\left(\lambda_{-}^{*}\right)+\left(1-\mu_{i}\right){\bar{\rho }}^{i}\left(\lambda_{+}^{*}\right).$$

Combing the two cases, we conclude that $\phi_{\lambda^{*}}=\left[n_{\lambda^{*},1},\ldots ,n_{\lambda^{*},N}\right]$ is $\lambda^{*}$-optimal under any $\mu_{i}\in \left[0,1\right]$. Hence, as long as the chosen $\mu_{i}$’s realize $\sum_{i=1}^{N}{\bar{\rho }}^{i}\left(\lambda^{*}\right)=M$, we can conclude that the randomized policy $\phi_{\lambda^{*}}$ is optimal for RP.

## Appendix L. Proof of Proposition 8

We notice that $M_{1}^{i}\left(\lambda^{*},-1\right)$ coincides with the decoupled model studied in Section 4.2. Therefore, we can use the results in Section 4.2 to prove the properties. Since the users share the same structure, we ignore the user index *i* for simplicity. According to the definition of $I_{x}$, we have

$$\begin{array}{cc} I_{x} & =\sum_{x^{\prime }}P_{x,x^{\prime }}\left(0\right)V\left(x^{\prime }\right)-\sum_{x^{\prime }}P_{x,x^{\prime }}\left(1\right)V\left(x^{\prime }\right)-\lambda^{*}\\ & =-\Delta V(x). \end{array}$$

Leveraging the results in the proof of Proposition 1, we have the following

- For state $x=(0,\hat{r})$, $I_{x}=-\lambda^{*}$.
- For state x=(s,0) where s>0, $I_{x}=-\lambda^{*}-p_{e}^{0}\left(1-2p\right)\omega$ where ω=(1−γ)[V(0,0)−V(s+1,0)]+γ[V(0,1)−V(s+1,1)]≤0.
- For state x=(s,1) where s>0, $I_{x}=-\lambda^{*}-\left(1-p_{e}^{1}\right)\left(1-2p\right)\omega$.

From the above three cases, we can easily conclude that $I_{x}\geq -\lambda^{*}$ and the equality holds when $\hat{r}=p_{e}^{0}=0$ or s=0. As is proven in Corollary 2, V(x) is non-decreasing in *s*. Hence, we can conclude that $I_{x}$ is also non-decreasing in *s*. To show that $I_{x}$ is monotone in $\hat{r}$, we consider two states $x_{1}=\left(s,1\right)$ and $x_{2}=\left(s,0\right)$. Then, we have

$$I_{x_{2}}-I_{x_{1}}=\Delta V\left(s,1\right)-\Delta V\left(s,0\right)=\left(1-p_{e}^{1}-p_{e}^{0}\right)\left(1-2p\right)\omega \leq 0.$$

Therefore, we can conclude that $I_{x}$ is non-decreasing in $\hat{r}$.

## Appendix M

**Algorithm A1** Improved Relative Value Iteration	
**Require:**	
	MDP M=(X,P,A,C)
	Convergence Criteria ϵ
1:	**procedure**RelativeValueIteration(M,ϵ)
2:	Initialize $V_{0}\left(x\right)=0$; ν=0
3:	Choose $x^{ref}\in X$ arbitrarily
4:	**while** $V_{\nu }$ is not converged (RVI converges when the maximum difference between the results of two consecutive iterations is less than ϵ) **do**
5:	**for** $x=(s,\hat{r})\in X$ **do**
6:	**if** ∃ active state $\left(s_{1},{\hat{r}}_{1}\right)$ s.t. $s_{1}\leq s$ and ${\hat{r}}_{1}\leq \hat{r}$ **then**
7:	$a^{*}\left(x\right)=1$
8:	$Q_{\nu +1}\left(x\right)=C\left(x,1\right)+\sum_{x^{\prime }}P_{xx^{\prime }}\left(1\right)V_{\nu }\left(x^{\prime }\right)$
9:	**else**
10:	**for** a∈A **do**
11:	$H_{x,a}=C\left(x,a\right)+\sum_{x^{\prime }}P_{xx^{\prime }}\left(a\right)V_{\nu }\left(x^{\prime }\right)$
12:	$a^{*}\left(x\right)=\arg\min_{a}\left\{H_{x,a}\right\}$
13:	$Q_{\nu +1}\left(x\right)=H_{x,a^{*}}$
14:	**for** x∈X **do**
15:	$V_{\nu +1}\left(x\right)=Q_{\nu +1}\left(x\right)-Q_{\nu +1}\left(x^{ref}\right)$
16:	ν=ν+1
	**return** $n\leftarrow a^{*}\left(x\right)$

**Algorithm A2** Bisection Search	
**Require:**	
	Maximum updates per transmission attempt *M*
	MDP $M_{N}\left(\lambda ,-1\right)=\left(X_{N},A_{N}\left(-1\right),P_{N},C_{N}\left(\lambda \right)\right)$
	Tolerance ξ
	Convergence criteria ϵ
1:	**procedure**BisectionSearch($M_{N}\left(\lambda ,-1\right)$, *M*, ξ, ϵ)
2:	Initialize $\lambda_{-}=0$; $\lambda_{+}=1$
3:	$\phi_{\lambda_{+}}\leftarrow \left(M_{N}\left(\lambda_{+},-1\right),\epsilon \right)$ using Section 5.1 and Proposition 6
4:	$\bar{\rho }\left(\lambda_{+}\right)\leftarrow \phi_{\lambda_{+}}$ using Proposition 2
5:	**while** $\bar{\rho }\left(\lambda_{+}\right)\geq M$ **do**
6:	$\lambda_{-}=\lambda_{+}$; $\lambda_{+}=2\lambda_{+}$
7:	$\phi_{\lambda_{+}}\leftarrow \left(M_{N}\left(\lambda_{+},-1\right),\epsilon \right)$ using Section 5.1 and Proposition 6
8:	$\bar{\rho }\left(\lambda_{+}\right)\leftarrow \phi_{\lambda_{+}}$ using Proposition 2
9:	**while** $\lambda_{+}-\lambda_{-}\geq 2\xi$ **do**
10:	$\lambda =\frac{\lambda_{+}+\lambda_{-}}{2}$
11:	$\phi_{\lambda }\leftarrow \left(M_{N}\left(\lambda ,-1\right),\epsilon \right)$ using Section 5.1 and Proposition 6
12:	$\bar{\rho }\left(\lambda \right)\leftarrow \phi_{\lambda }$ using Proposition 2
13:	**if** $\bar{\rho }\left(\lambda \right)>M$ **then**
14:	$\lambda_{-}=\lambda$
15:	**else**
16:	$\lambda_{+}=\lambda$
	**return** $\left(\lambda_{+}^{*},\lambda_{-}^{*}\right)\leftarrow \left(\lambda_{+},\lambda_{-}\right)$
